# Oxidative Activation of the Heme Nitric Oxide/Oxygen-Binding Protein (H-NOX) from 

**DOI:** 10.1021/acs.biochem.5c00262

**Published:** 2025-07-28

**Authors:** Aishat Alatishe, Therese Albert, Cameron Christopher Lee-Lopez, Rashedul Hasan, Pierre Moënne-Loccoz, Kelly N. Chacón, Erik T. Yukl

**Affiliations:** † Department of Chemistry and Biochemistry, 4423 New Mexico State University, Las Cruces, New Mexico 88003, United States; ‡ Department of Chemical Physiology and Biochemistry, School of Medicine, 6684 Oregon Health & Science University, Portland, Oregon 97239, United States; § Department of Chemistry, 6686 Reed College, Portland, Oregon 97202, United States

## Abstract

The heme nitric oxide/oxygen-binding proteins (H-NOX) are bacterial homologues of the sensor domain of mammalian soluble guanylate cyclase (sGC), a multidomain enzyme that catalyzes the production of cyclic guanosine monophosphate (cGMP) in response to NO. In facultative anaerobes, H-NOX proteins sense nitric oxide (NO) and regulate various communal behaviors including biofilm formation, motility, virulence, and quorum sensing. Rupture of the proximal heme iron-histidine bond during the formation of a five-coordinate low-spin ferrous nitrosyl (5cLS Fe­(II)-NO) heme is thought to be required for H-NOX activation, allowing them to interact with downstream signaling partners such as diguanylate cyclases (DGC), phosphodiesterases (PDE), or histidine kinases (HK). Some H-NOX homologues also contain a conserved Cys-ligated zinc-binding site, which can respond to oxidative stress, at least *in vitro*. Although classified as an obligate aerobe, encodes an apparent NO-sensing *hnox* gene adjacent to that of the HK gene *hnok*. Spectroscopic analysis of the *Cc* H-NOX protein reveals characteristics similar to those of other NO-sensing H-NOX homologues, including the formation of a 5cLS Fe­(II)-NO heme. Surprisingly, this form is completely noninhibitory to HnoK autophosphorylation, in contrast to what has been observed for every other related system to date. Rather, oxidation of the zinc ligand Cys residues activates *Cc* H-NOX. X-ray absorption fine structure (EXAFS) data reveal a change in zinc coordination upon oxidation but no loss of zinc. This work illustrates the breadth of H-NOX-signaling mechanisms and expands our understanding of signaling pathways in which this widespread protein participates.

## Graphical abstract

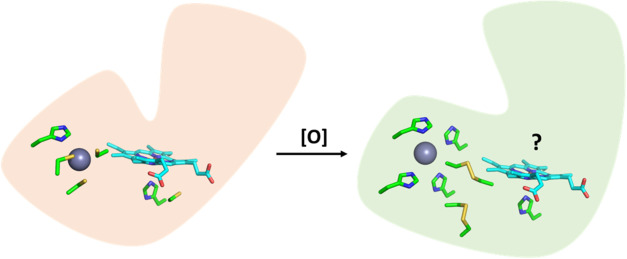

## Introduction

Heme nitric oxide/oxygen-binding (H-NOX) domains are widespread among bacteria, where they play important roles in mediating behavioral responses to changing environments. They can be functionally divided into two groups based on the lifestyle of the bacteria from which they originate.^1^ Those found in strict anaerobes form stable Fe­(II)-O_2_ species through hydrogen bonding to a conserved Tyr residue in the distal pocket (*e.g*., Y140 in H-NOX).^2^ They are fused to methyl-accepting chemotaxis proteins (MCP) and likely mediate chemotaxis in response to O_2_, which is toxic to these organisms. In contrast, the H-NOX proteins from facultative anaerobes lack the distal Tyr residue and do not stably bind to O_2_. They modulate the activity of histidine kinases (HK) or H-NOX-associated cyclic-di-GMP processing enzymes (HaCE) in a NO-dependent manner, regulating biofilm formation,^3–6^ symbiont/host colonization^7^ or quorum sensing.^8^

Well-studied examples of NO-responsive H-NOX proteins include those from and . Both proteins in the Fe­(III) or Fe­(II)-NO states strongly inhibit autophosphorylation of the HK HnoK,^9,10^ which itself phosphorylates the c-di-GMP phosphodiesterase HnoB, activating it to hydrolyze c-di-GMP. Thus, in , H-NOX activation by NO stimulates biofilm formation,^5^ presumably by elevating c-di-GMP levels. Both proteins also share a conserved zinc-binding motif consisting of 3 Cys and 1 His/Gln residue.^11^ Oxidation of these Cys residues to form disulfide bonds has been demonstrated to activate *Vc* H-NOX even when heme is not bound.^12^ Although the ligation state changes, zinc remains bound to oxidized H-NOX, suggesting that oxidant sensing in heme-free *Vc* H-NOX proceeds via specific disulfide bond formation and reorganization of the zinc site.

A phylogenetic analysis of approximately 600 homologues of *Vc* H-NOX showed that these sequences clustered into 4 groups, depending on the presence of this zinc site.^12^ The 3 Cys, 1 His/Gln motif is absent in group 1, conserved in groups 2 and 3, and a different conserved motif of 3 Cys residues is present in group 4. Group 2 includes H-NOX proteins from and , which have been confirmed to bind zinc (Figure S1). Interestingly, group 3 includes an H-NOX homologue from (aka ), an obligate aerobe adapted to nutrient-poor freshwater environments that is not known to denitrify.^13^ This lifestyle begs the question of why such an organism would require an NO sensor, as it is unexpected to encounter either exogenous or endogenous sources of NO. Further, deletion of the *hnox* gene in disrupted cell-cycle progression and surface layer formation even in the absence of NO.^14^ Thus, whether *Cc* H-NOX acts as an NO sensor or whether it even binds heme is unknown. In fact, to our knowledge, none of the H-NOX proteins from group 3 have been characterized to date.

*Cc* H-NOX is encoded on a gene adjacent to that of an HK, herein referred to as *hnoK*. *Cc* HnoK shares homology to a group of kinases called H-NOX-associated quorum-sensing kinases (HqsK) in^8^ and .^15^ In addition to the canonical HK domain, these proteins include a C-terminal response regulatory (RR) domain with a conserved Asp residue. The flow of phosphotransfer is proposed to proceed from autophosphorylation at the conserved His of the HK domain to the conserved Asp of the RR domain. In the cited *Vibrio* species, the phosphoryl group is then transferred to the phosphotransfer protein LuxU, part of a phosphorelay system regulated by quorum sensing.^16,17^ H-NOX in the NO-bound form but not in the Fe­(II) form inhibits HqsK autophosphorylation activity, thereby inhibiting this phosphotransfer pathway. has no LuxU homologue, but it does encode a quorum-sensing phosphorelay system (CckA-ChpT-CtrA) that regulates cell-cycle progression in response to c-di-GMP.^18^ Our previous study has hinted at the participation of H-NOX/HnoK signaling in this pathway,^14^ but no direct targets of HnoK phosphorylation have yet been identified. Nevertheless, the reasonable prediction would be that *Cc* H-NOX binds NO and inhibits HnoK autophosphorylation, as has been observed for all of the H-NOX/HK systems studied to date.

Here, we describe the spectroscopic and functional characterization of H-NOX from . Stoichiometric heme and zinc binding were confirmed, and interactions between gaseous ligands and the protein were investigated by ultraviolet–visible (UV–vis), resonance Raman (rR), and electron paramagnetic resonance (EPR) spectroscopy. Remarkably, despite spectroscopic characteristics that were mostly typical for NO-sensitive H-NOX proteins, NO binding failed to activate *Cc* H-NOX to inhibit HnoK autophosphorylation. Only oxidation of the Cys residues at the zinc site was capable of converting *Cc* H-NOX to a form that could inhibit HnoK autophosphorylation. X-ray Absorption Fine Structure (EXAFS) analysis confirmed a change in zinc coordination upon oxidation analogous to that observed for *Vc* H-NOX.^12^ To the best of our knowledge, this is the first observation of an inactive Fe­(II)-NO state for an H-NOX protein of this class. This observation has important implications for the evolution of the H-NOX protein family and the environmental signals to which they respond.

## Materials and Methods

### Expression and Purification of Proteins

The entire *hnox* gene (Uniprot ID: A0A0H3CDX0_CAUVN) was amplified by polymerase chain reaction (PCR) from CB15 genomic DNA. The PCR product was cloned into a pCDFDuet-1 vector (Novagen) at the NdeI restriction site using the Gibson cloning method.^19^ A 6-His tag was added to the N-terminus of the expressed protein by site-directed mutagenesis. For the coexpression of H-NOX with HnoK, the entire cassette encompassing the overlapping *hnox* and *hnok* (Uniprot ID: A0A0H3CAQ8_CAUVN) genes was amplified and cloned into the *Bam*HI restriction site of the pCDFDuet-1 vector (Novagen), resulting in an N-terminal 6-His-tagged H-NOX and untagged HnoK. WT HnoK alone was cloned into pCDFDuet at the *Bam*HI restriction site to yield a N-terminal 6-His-tagged construct, as well as into a pMAL-c5x plasmid (New England Biolabs) using a *Bam*HI restriction site to yield an N-terminal fusion of HnoK with maltose-binding protein (MBP). The D543N mutation of HnoK in each case was generated by site-directed mutagenesis. Sequences for all plasmids used in this study have been added to the Supporting Information as downloadable GenBank (.gnbk) files.

Expression plasmids were transformed into BL21 DE3 cells and cultured in LB medium with 50 mg/mL streptomycin or 100 mg/mL ampicillin at 37 °C and 220 rpm until reaching an optical density of 0.4 at 600 nm. To induce overexpression, IPTG was added to 0.4 mM, and the temperature was lowered to 18 °C while the cells were cultivated with shaking overnight. The cells were collected by centrifugation at 4000*g* for 30 min at 4 °C.

Cell pellets were resuspended in lysis buffer containing 20 mM Tris, pH 8, 150 mM NaCl, and lysed using a homogenizer (Avestin, Inc.). Cell debris was removed by centrifugation at 25,000g for 30 min at 4 °C. For His-tagged proteins, imidazole was added to 10 mM, and the lysate was applied directly to a Ni-NTA column equilibrated with lysis buffer containing 10 mM imidazole. The column was washed with 10 column volumes of lysis buffer containing 10 mM imidazole, followed by 3 column volumes of lysis buffer containing 20 mM imidazole. Protein was eluted with 250 mM imidazole. For MBP fusion proteins, the lysis buffer lacked imidazole, and the lysate was applied to an amylose column (New England Biolabs), washed with lysis buffer, and eluted with lysis buffer containing 10 mM maltose. Finally, the samples were concentrated and applied to a HiPrep Sephacryl S-200 HR column (Cytiva) equilibrated with lysis buffer. Elution volumes for H-NOX and HnoK were consistent with their existence as monomer and homodimer, respectively. At this point, proteins were judged sufficiently pure by sodium dodecyl sulfate–polyacrylamide gel electrophoresis (SDS–PAGE) (Figure S2).

The Bradford assay^20^ seemed to consistently overestimate H-NOX protein concentration in comparison to heme and zinc concentrations (see Results Section). To verify and correct for this, a sample of purified H-NOX at 3.0 mg/mL, as determined by the Bradford assay, was submitted for total amino acid analysis (AAA) (Creative Proteomics), generally considered the gold standard for protein quantitation. The AAA results produced a total protein concentration of 1.89 mg/mL for this sample, indicating a correction factor of 1.6.

### Absorbance Spectroscopy

All spectra were collected on a Cary 60 UV–vis spectrophotometer (Agilent). The Fe­(II) form could be generated by anaerobic addition of DTT to 50 mM or sodium dithionite to 1 mM to nitrogen-purged H-NOX in a septum-sealed cuvette. Formation of a stable Fe­(II)-O_2_ complex was assessed by exposure of the DTT-reduced sample to air and recording changes in the absorbance spectrum over time. The Fe­(II)-CO form could be generated by the addition of CO gas to the Fe­(II) form. Stock solutions of diethylamine NONOate (DEA-NONOate) (Cayman Chemicals) were made in 10 mM NaOH and quantified using the extinction coefficient ε_250_ = 6500 M^–1^ cm^–1^. The Fe­(II)-NO form was generated by anaerobic addition of a nitrogen-purged DEA-NONOate solution to 1.0 mM to the as-isolated Fe­(III) form and allowed to incubate for 1 h. Alternatively, NONOate could be added to the Fe­(II) form generated as described above. Oxidation of H-NOX thiols was performed by incubation of the protein with 1.0 mM sodium hypochlorite (NaOCl) or diamide for 1 h. The concentration of sodium hypochlorite was determined from its extinction coefficient ε_292_ = 350 M^–1^ cm^–1^.^21^ The extinction coefficient of the as-isolated Fe­(III) form was determined by pyridine hemochrome assay^22^ on three independent preparations of protein. Extinction coefficients for the different heme oxidation and coordination states were determined relative to the average value of the as-isolated Fe­(III) form.

### H-NOX Sample Preparation

In each case, H-NOX was treated at a concentration of 100–200 μM and diluted to the desired concentrations for autophosphorylation assays. Fe­(II)-NO H-NOX was generated by anaerobic incubation with NONOate as described above. The solution was briefly purged with nitrogen to remove excess NO, and DTT was added to 1 mM to maintain reduced thiol groups. A blank of purged buffer with added NONOate was made simultaneously and added to autophosphorylation assays (see below) to confirm that neither NO nor its oxidation products alone inhibit HnoK. The oxidized forms of H-HOX were generated by incubation with 1.0 mM NaOCl or diamide for 1 h at room temperature, followed by desalting into lysis buffer using Zeba desalting columns (Pierce). Protein concentrations were measured after the final desalting steps using the Bradford assay and were subsequently corrected using data from amino acid analysis. The stability of all H-NOX solutions was confirmed for over 1 h based on absorbance spectra.

### Resonance Raman Spectroscopy

Resonance Raman spectra were collected on a McPherson spectrograph equipped with a liquid-nitrogen-cooled CCD camera (LN1100PB, Princeton Instrument) using a 407 nm laser excitation from a Krypton laser (Innova 302C, Coherent) on solutions at ∼100 μM protein concentrations. Room-temperature spectra were obtained using a 90° geometry, while a backscattering geometry was used on samples maintained at 110 K. The ferrous protein was prepared by the addition of 1 mM dithionite. Excess reductant was removed using desalting spin columns inside an anaerobic glovebox (Omnilab system, Vacuum Atmosphere Co.) before exposure to CO or NO gas (∼0.1 Atm) in Eppendorf tubes sealed with septa and transferred to Raman capillaries before freezing in liquid nitrogen. Isotopically labeled gases were purchased from the Cambridge Isotope Laboratory.

### Fourier Transform Infrared (FTIR) Spectroscopy

Low-temperature FTIR spectra were collected on samples sandwiched between two CaF_2_ windows separated by a 15 μm spacer (International Crystal Laboratories, Garfield, NJ). Carbonyl complexes were prepared inside the glovebox, preincubating the dithionite-reduced protein with CO inside a glass vial before depositing a 25 μL droplet of protein solution on the bottom window, before gently dropping the second window on the sample and clamping the cell. After mounting the cell to a closed-circuit cryostat (Displex, Advanced Research Systems), samples were brought to 30 K inside the sample chamber of the FTIR spectrograph. Spectra were collected on a Bruker Vertex 80 equipped with a liquid-N_2_-cooled MCT detector and purged with compressed dried air depleted of CO_2_ (purge gas generator, Puregas LLC). Sets of 2000-scan accumulations were collected at a 4 cm^–1^ resolution. After acquisition of “dark” spectra, photolysis of the carbonyl complexes was performed through illumination of the samples with a 300W arc lamp with filtering of UV and near-infrared emissions for 1 min before collecting a series of illuminated spectra.

### Electron Paramagnetic Resonance Spectroscopy

EPR spectra were collected on a Bruker E500 spectrometer equipped with a SuperX microwave bridge, a superhigh Q cavity, and a liquid-nitrogen cooled nitrogen flow cryostat. Spectra were fitted using EasySpin.^23^

### Metal Content Analysis

H-NOX protein in various forms was diluted to 15 μM in 4 M HNO_3_ for overnight digestion at 70 °C. Prior to metal analysis, samples were diluted 10-fold with deionized water. Samples were analyzed on a PerkinElmer 2100 DV inductively coupled plasma-optical emission spectrometer (ICP-OES), calibrated with a multielement standard. The wavelengths for measuring Fe and Zn were 238.204 and 213.857 nm, respectively. Samples and standards were measured in triplicate.

### Assays for Cysteine Thiol

H-NOX was used as-isolated or treated with 1 mM DTT, diamide, or sodium hypochlorite for 1 h, followed by desalting into lysis buffer. Each sample was diluted to 20 μM in 100 mM K_2_HPO_4_, pH 7.0, 1.0 mM EDTA, and 6 M guanidine hydrochloride. An absorption spectrum was collected, and 5,5′-dithio-bis-[2-nitrobenzoic acid] (DTNB) was added to 0.08 mg/mL and incubated for 15 min at room temperature. The concentration of free thiol was measured by subtracting the spectrum of the protein acquired before DTNB addition from that acquired afterward using ε_412_ = 14,150 M^–1^ cm^–1^.^24,25^

### Autophosphorylation Assays

The kinase activity of HnoK was assayed by the addition of 2 μCi ATP [γ-^32^P], 1 mM ATP, and 5 mM MgCl_2_ or MnCl_2_ to 20 μM HnoK in lysis or HEPES buffer with or without 1 mM DTT at room temperature. Aliquots were quenched after 60 or 120 min by incubation in 4× Laemmli sample loading buffer for approximately 20–40 min at room temperature. SDS–PAGE was performed using 4–15% tris-glycine gels (Bio-Rad) run at 200 mV for 40 min. Gels were dried at 80 °C for 1 h on a slab gel dryer. The dried gels were exposed overnight (16–24 h) on a Kodak phosphorimaging plate, imaged using a Storm Phosphorimager (Amersham) at 100 μm resolution, and quantified using ImageJ.^26^ For H-NOX inhibition assays, H-NOX in different forms and various concentrations was included in the HnoK assay mixtures and incubated for 1 h prior to quenching and SDS–PAGE. For the Fe­(II)-NO H-NOX experiments, NONOate-treated buffer was included as a control at a volume equivalent to that of the largest volume of Fe­(II)-NO H-NOX added. For experiments with oxidized H-NOX, 1 mM DTT was included in the highest H-NOX concentration sample to determine whether oxidation and HnoK inhibition are reversible.

### X-Ray Absorption Fine Structure (EXAFS)

A 500 μL portion of H-NOX at approximately 125 μM heme concentration was incubated with 1 mM DTT or 1 mM sodium hypochlorite in lysis buffer for 1 h at room temperature, followed by desalting. The samples were concentrated to ∼80 μL using centrifugal filtration devices (Millipore). 80 μL of protein or buffer was combined with 20 μL of ethylene glycol, loaded into EXAFS cuvettes, and flash frozen in liquid nitrogen. The heme states and final heme concentrations were confirmed by absorbance spectroscopy at 474 and 534 μM for the reduced and oxidized samples, respectively.

X-ray absorption data were collected at Stanford Synchrotron Radiation Lightsource. Extended X-ray absorption fine structure (EXAFS) of Zn (9658 eV) was measured on beamlines 9–3 and 7–3 in duplicate when available using a Si 220 monochromator with crystal orientation φ = 0° to reduce the likelihood of known crystal glitches in the Zn energy range. Samples were measured as frozen aqueous glasses in 20% ethylene glycol at 15 K, and the X-ray absorbance was detected as Kα fluorescence using either a 100-element (beamline 9–3) or 30-element (beamline 7–3) Canberra Ge array detector. A Z-1 metal oxide filter (Cu) and Soller slit assembly was placed in front of the detector to attenuate the elastic scatter peak. Four to six scans of a buffer blank were measured at the absorption edge and subtracted from the raw data to produce a flat pre-edge and eliminate residual Cu Kβ fluorescence of the metal oxide filter. Energy calibration was achieved by placing a Zn metal foil between the second and third ionization chambers. Data reduction and background subtraction were performed using EXAFSPAK.^27^ The data from each detector channel were inspected for dropouts and glitches before being included in the final average. EXAFS simulation was carried out using the program EXCURVE 9.2 as previously described.^28–30^

### Circular Dichroism (CD) Spectroscopy

Circular dichroism (CD) spectra were recorded at 20 °C by using a Jasco-1500 spectropolarimeter with a temperature-regulated cuvette chamber. H-NOX, as-isolated or treated with 1 mM NaOCl for 1 h, was diluted to 5 μM in 5 mM K_2_HPO_4,_ pH 8.0, 30 mM NaCl in a 1 mm quartz cuvette. Spectra were acquired from 180 to 260 at a 1 nm bandwidth, 2 s response time, 0.5 nm data pitch, and 10 nm/min scan speed. Baseline and sample spectra are the average of three accumulations, and units have been converted to mean residue ellipticity. Spectral fitting for secondary structural analysis was performed with the Bestsel web server.^31–33^

## Results

### *Cc* H-NOX Characterization

Initial attempts to purify *Cc* H-NOX were met with limited success due to poor solubility in the heterologous host. In an attempt to improve yields, H-NOX was coexpressed with its presumptive interaction partner HnoK. These two genes have overlapping reading frames, so the entire cassette was cloned into the first multiple cloning site of the pCDF-DUET vector, resulting in expression of H-NOX with an N-terminal 6-His tag along with the untagged HnoK. Protein was purified by nickel affinity chromatography followed by size exclusion chromatography (SEC) to isolate free H-NOX.

Three independent preparations of *Cc* H-NOX were analyzed by the pyridine hemochrome method to determine heme extinction coefficients and ICP-OES to quantify both iron and zinc levels. Both iron and zinc were substoichiometric at 0.63 ± 0.05 equiv and 0.61 ± 0.01 equiv, respectively, using protein concentration as determined by Bradford assay. However, the fact that both values were very consistent between preps, and with one another, suggests that these are homogeneous H-NOX preparations with one zinc and one heme bound per protein, and that the Bradford assay substantially overestimates the protein concentration. Indeed, total amino acid analysis indicated that the Bradford assay overestimates the H-NOX concentration by 1.6-fold. Applying this correction factor results in iron and zinc contents of 1.01 ± 0.07 and 0.98 ± 0.02 equiv, respectively.

*Cc* H-NOX as-isolated exhibited a UV–vis spectrum with a Soret maximum at 385 nm and a broad feature around 650 nm (Figure 1 and Table 1), suggestive of a high-spin Fe­(III) species. The reactivity of *Cc* H-NOX toward reducing agents, oxidizing agents, and exogenous ligands NO and CO was assessed by UV–vis (Table 1 and Figure 1). The Fe­(II) state could be achieved by anaerobic incubation of the protein with high concentrations of DTT (>10 mM) or low concentrations (<1 mM) of sodium dithionite. The reduced state reacted slowly with oxygen but with no suggestion of the formation of a stable Fe­(II)-O_2_ state. Rather, ferrous heme signals decayed over several hours of exposure to air (Figure S3). Addition of sodium hypochlorite (NaOCl) to resting Fe­(III) H-NOX resulted in a shift of the Soret peak from 385 to 414 nm and disappearance of the peak around 650 nm, suggesting the formation of a six-coordinate low-spin (6cLS) Fe­(III) heme. The Fe­(II)-NO species could be prepared by the anaerobic addition of NO, from gas or the NONOate donor, to either the Fe­(III) or Fe­(II) states. The Soret maximum below 400 nm indicates that the Fe­(II)-NO heme exists predominantly as a 5-coordinate low-spin (5cLS) species consistent with most other H-NOX proteins (see Table 3). Finally, the Fe­(II)-CO state was achieved by the addition of CO gas to the Fe­(II) state and exhibits typical 6cLS features.

**Figure 1:**
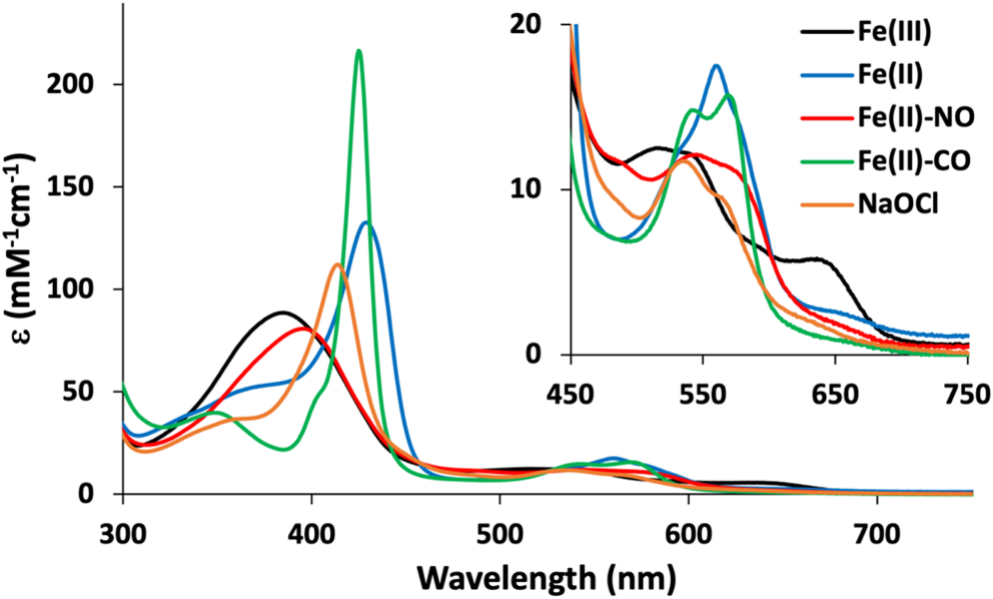
UV–vis absorption spectra for various forms of *Cc* H-NOX.

**Table 1:** Absorption Characteristics of Various Forms of *Cc* H-NOX

	λ_Max_ (nm)	ε (mM^–1^ cm^–1^)
H-NOX Fe(III)	385	88.7 ± 5.0^a^
H-NOX Fe(II)	429	133
H-NOX Fe(II)-NO	395	81
H-NOX Fe(II)-CO	425	217
H-NOX Fe(III) NaOCl	414	112

a Experimental values determined by pyridine hemochrome assay. Uncertainties are given as standard deviations (*n* = 3 independent protein preparations). Other values were determined relative to the average Fe­(III) value.

### Resonance Raman

The high-frequency regions of the rR spectra of Fe­(III) and Fe­(II) *Cc* H-NOX obtained with Soret excitation at room temperature are shown in Figure 2. The porphyrin skeletal modes ν_4_, ν_3_, ν_2_, and ν_10_ are observed at frequencies characteristic of 5-coordinate high-spin (5cHS) Fe­(III) and 5cHS Fe­(II) heme complexes.^34^ With excitation at 442 nm, the low-frequency spectrum of the reduced protein shows a strong band at 212 cm^–1^, characteristic of a ν­(Fe–N_His_) mode from a neutral proximal His ligand.^35^

**Figure 2:**
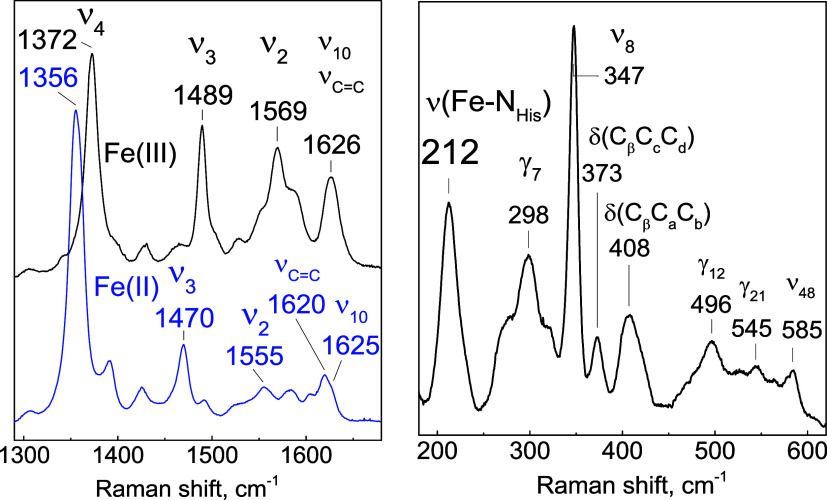
Room-temperature rR spectra of *Cc* H-NOX. (left) High-frequency region for as-isolated Fe­(III) (black) and Fe­(II) (blue) states (λ_exc_ = 406 nm). (right) Low-frequency region of the Fe­(II) state (λ_exc_ = 442 nm).

Resonance Raman spectra of the carbonyl complexes were acquired at a low temperature (110 K) due to the photolability of the CO ligand (Figure 3). The low-frequency region showed two bands at 481 and 495 cm^–1^, which downshift approximately 3 cm^–1^ upon ^13^CO substitution, identifying them as two distinct ν­(Fe-CO) modes. The Fe–C–O bending mode is identified at 570 cm^–1^ as it downshifts to 16 cm^–1^ with ^13^CO substitution. Low-temperature FTIR photolysis difference spectra identify a dominant ν­(C–O) mode at 1988 cm^–1^ that downshifts to 45 cm^–1^ upon ^13^CO substitution. These observed frequencies are characteristic of heme-carbonyl complexes with a neutral histidine coordinating *trans* to the CO group^36^ (Table 3 and Figure 8).

**Figure 3:**
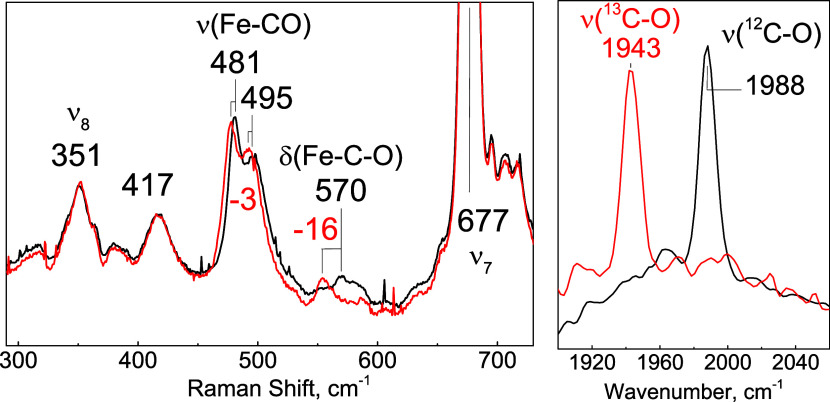
Spectra of the Fe­(II)-CO *Cc* H-NOX. (left) rR spectra of the low-frequency region and (right) light-induced FTIR difference spectra in the high-frequency region comparing ^12^CO (black) and ^13^CO samples (red).

Low-temperature rR spectra of the Fe­(II)-NO complex identified a band at 532 cm^–1^ that downshifted to 523 cm^–1^ upon substitution with ^15^NO (Figure 4). This 532 cm^–1^ frequency is typical of a ν­(Fe-NO) mode for a 5cLS Fe­(II)-NO complex, consistent with the UV–vis spectrum and other H-NOX proteins (Table 3). The corresponding ν­(N–O) mode could not be identified in the high-frequency region of the rR spectra, as these modes are buried under intense heme modes and the rising background commonly observed with frozen protein solutions. The EPR spectrum of the Fe­(II)-NO complex collected at 134 K is dominated by a *g* ∼ 2 signal with a distinctive three-line ^14^N-hyperfine structure (*A*_NO_ = 17 G) at the low-field negative feature characteristic of 5-coordinate nitrosyl species (Figure 4). Best fits require a rhombic pattern with *g*, A, and *g* strain values that closely resemble values observed with 5cLS nitrosyl complexes of sGC^37^ and *Vc* H-NOX.^38^

**Figure 4:**
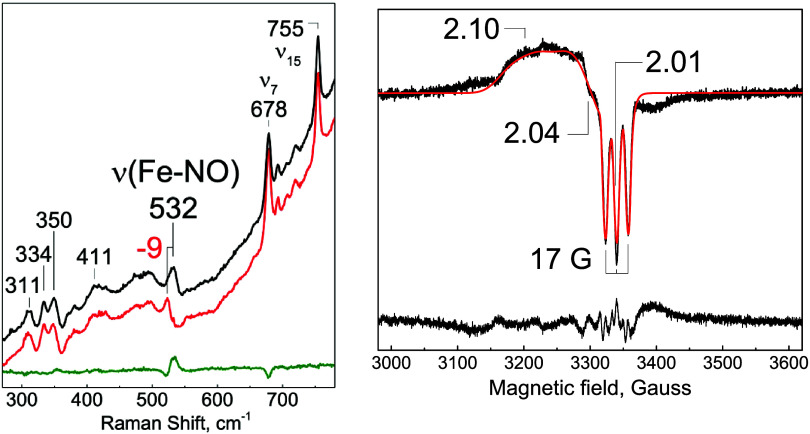
Spectra of Fe­(II)­NO *Cc* H-NOX. (left) Low-temperature rR spectra of Fe­(II)-NO complexes prepared with unlabeled (black) and ^15^N-labeled (red) NO gas and the ^14^NO–^15^NO difference spectrum (green). (right) EPR spectrum of the Fe­(II)-NO complex (top, black) with an overlapping fit (red) and residual (bottom, black). Experimental conditions: protein concentration, 40 μM; temperature 120 K; microwave power, 2 mW; microwave frequency, 9.40 GHz; modulation frequency, 100 kHz; modulation amplitude, 6 G (fitting parameters: *g* values = 2.098, 2.037, 2.012; *g* strain = 0.030, 0.030, 0.001; *A* values = 76, 50, 49 MHz).

### HnoK Autophosphorylation

We could observe no autophosphorylation for WT HnoK. This is most likely due to the presence of an RR domain whose conserved Asp residue (D543) may be phosphorylated by the HK domain. The resulting phosphor-Asp residue is unstable,^39^ and the signal is lost. Similar results were observed for related HK-RR proteins regulated by H-NOX in^8^ and .^15^ Thus, we generated a N-terminal 6-His construct of D543N HnoK for initial autophosphorylation optimization experiments in the absence of H-NOX. This enzyme exhibited a strong preference for manganese over magnesium (Figure S4). This is unusual among histidine kinases but not unprecedented,^40,41^ and it may relate to the preferred geometry of M^2+^-ATP binding. HnoK activity did not respond significantly to the inclusion of 1 mM DTT. We subsequently expressed and purified a maltose-binding protein fusion (D543N HnoK-MBP), which exhibited dramatically improved yields and improved autophosphorylation activity (Figure S4). For these reasons, D543N HnoK-MBP was used for the autophosphorylation assays shown (Figure 5A,B). A second, smaller band can be observed for this construct with an MW similar to that of the 6-His construct (Figures S2 and S4C). This is likely an untagged HnoK generated by a small amount of autoproteolysis of the linker between MBP and HnoK. Importantly, its autophosphorylation intensity mirrors that of the fusion protein, indicating that it is also subject to regulation by H-NOX. Similarly, the 6-His construct showed the same patterns of inhibition as HnoK-MBP (Figure S5).

**Figure 5:**
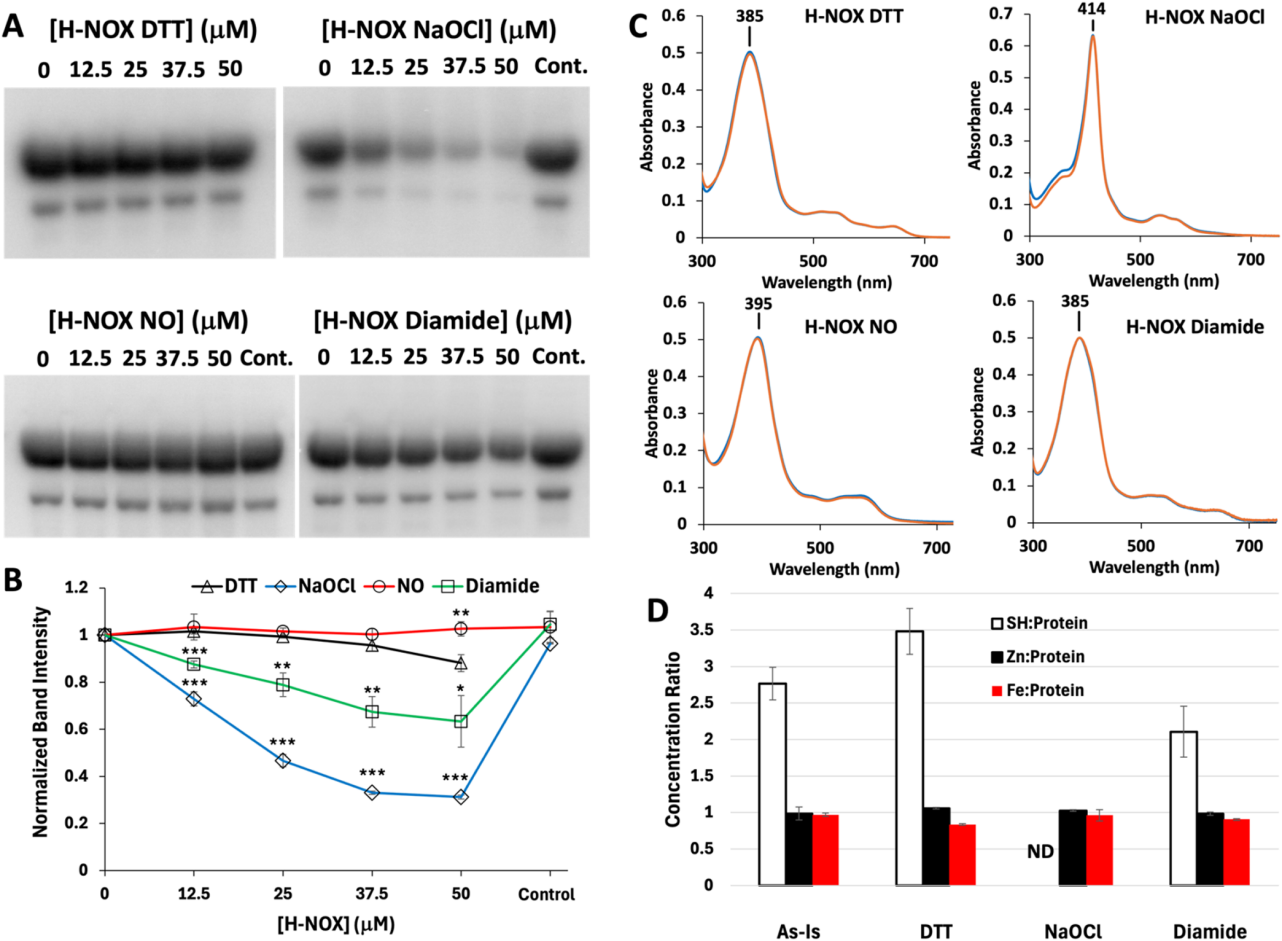
Inhibition of HnoK autophosphorylation by *Cc* H-NOX. (A) Representative autoradiography of HnoK autophosphorylation in the presence of increasing concentrations of H-NOX in various states. (B) Quantitation of HnoK autophosphorylation. Error bars represent the standard deviation from two replicate experiments. “Control” refers to the addition of 1 mM DTT to the highest concentration of diamide- and NaOCl-treated H-NOX samples or the addition of NONOate-treated buffer to HnoK alone in the H-NOX NO data series (see Materials and Methods Section). Statistical significance determined by multiple unpaired *t-test* comparisons against the DTT sample at each concentration (* *p* < 0.1, ** *p* < 0.05, ***p < 0.01). (C) UV–vis spectra for all H-NOX states immediately before use in autophosphorylation experiments (blue) and after 90 min incubation at room temperature (orange). (D) Quantitation of total protein thiol (white bars), zinc (black bars), and iron (red bars) for H-NOX as-isolated (As-Is) or after various treatments given in molar ratio of analyte to protein. Error bars represent the standard deviation from two replicate experiments. ND = Not Detected.

In these experiments, we hoped to detangle the influence of the heme state of H-NOX from its Cys oxidation on HnoK inhibition. Thus, we assessed HnoK autophosphorylation inhibition by 5cHS Fe­(III) (“H-NOX DTT” in Figure 5) and Fe­(II)-NO H-NOX (“H-NOX NO” in Figure 5) in the presence of 1 mM DTT, which has no effect on their heme electronic spectra (Figure 5C) but will maintain Cys residues in the reduced state. Oxidation of Cys was achieved by incubation with 1 mM sodium hypochlorite (“H-NOX NaOCl” in Figure 5) or 1 mM diamide (“H-NOX Diamide” in Figure 5) for 1 h at room temperature, followed by oxidant removal by desalting. The former converts the heme to a 6cLS Fe­(III) state, while the latter has no effect on the 5cHS Fe­(III) heme spectrum (Figure S6). The oxidation state of Cys and the stability of the heme forms under the experimental conditions were confirmed by DTNB assay (Figure 5D) and UV–vis spectrophotometry (Figure 5C), respectively. We also measured iron and zinc levels in each H-NOX sample by ICP-OES (Figure 5D) and confirmed no significant loss of either metal under any condition.

The autoradiography results show that the presence of 1 mM DTT prevented any inhibition of HnoK autophosphorylation, irrespective of the heme state. All H-NOX heme types were stable in the presence of 1 mM DTT with the exception of the 6cLS Fe­(III) generated by treatment with sodium hypochlorite, which appears to undergo reduction and heme degradation (Figure S7). Thus, it is unclear whether this is responsible for the loss of HnoK inhibition in the presence of DTT or if this is due to Cys reduction. However, diamide treatment does not affect the 5cHS Fe­(III) heme state, but it results in partial oxidation of Cys and partial inhibition of HnoK, which is reversible by the addition of DTT. This strongly suggests that Cys oxidation state, rather than heme state, is the primary determinant of HnoK inhibitory activity by *Cc* H-NOX.

### Zinc EXAFS of *Cc* H-NOX DTT and H-NOX NaOCl

Zinc K-edge EXAFS was employed to experimentally define the coordination environment of zinc in H-NOX treated with 1 mM DTT or 1 mM NaOCl as described above. The Fourier transform (FT) of the reduced form (Figure 6A) shows a strong peak centered around 2.310 Å, consistent with sulfur ligation, with a slight shoulder at 2.010 Å due to oxygen or nitrogen ligation. The simulated EXAFS of the reduced form (Figure 6A inset) gave an excellent fit to the data and indicates 3 S and 1 O/N ligands (Table 2), consistent with coordination from Cys138, Cys163, Cys171, and His160, which are conserved zinc ligands in the structure of H-NOX.^11^ Of note are the low Debye–Waller (DW) values for all four ligands, indicating that these residues do not exhibit notable flux upon binding of the metal ion. In contrast, the EXAFS of oxidized H-NOX (Figure 6B) was significantly different and exhibited weaker scattering atoms at shorter distances, which is consistent with a change from a primarily sulfur ligation sphere to one dominated by multiple scattering nitrogen ligands. These contributions were best modeled by an all-His environment, confirming the loss of zinc binding from the sulfur-dominated site upon oxidation.

**Figure 6:**
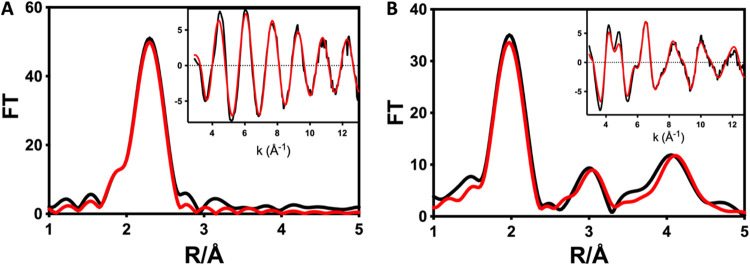
Zn K EXAFS and Fourier transforms of DTT-reduced (A) and NaOCl-oxidized (B) H-NOX. Black traces represent experimental data, and red traces are simulations. Parameters used to generate the simulated spectra are given in Table 2.

**Table 2:** Fit Parameters for the Zn EXAFS Simulations

	Fl	no	*R* (Å)	DW (Å^2^)	no	*R* (Å)	DW (Å^2^)	no	*R* (Å)	DW (Å^2^)	*E*_0_
Nn K		Zn–S			Zn–N/O			Zn–N(His)			
red	0.33	3	2.310	0.005	1	2.010	0.003				–11.48
Ox	0.37							4	1.993	0.006	

### Circular Dichroism Spectroscopy

Circular dichroism (CD) spectroscopy was employed to evaluate changes in the *Cc* H-NOX secondary structure that may accompany oxidation (Figure 7). The H-NOX protein was used either as-isolated or after incubation with 1 mM NaOCl. Conversion from the 5cHS Fe­(III) to 6cLS Fe­(III) was confirmed directly by UV–vis of the CD sample. CD spectra were normalized according to peptide bond absorbance at 220 nm. Both spectra exhibit two negative peaks around 210 and 220 nm, characteristic of α helical character, consistent with known H-NOX structures. However, a difference spectrum of the as-isolated minus the oxidized sample shows the loss of CD features upon oxidation. A fit to the difference spectrum suggests that the secondary structure lost upon oxidation is of predominantly antiparallel β sheet character (45%) with virtually no α helical contribution.

**Figure 7:**
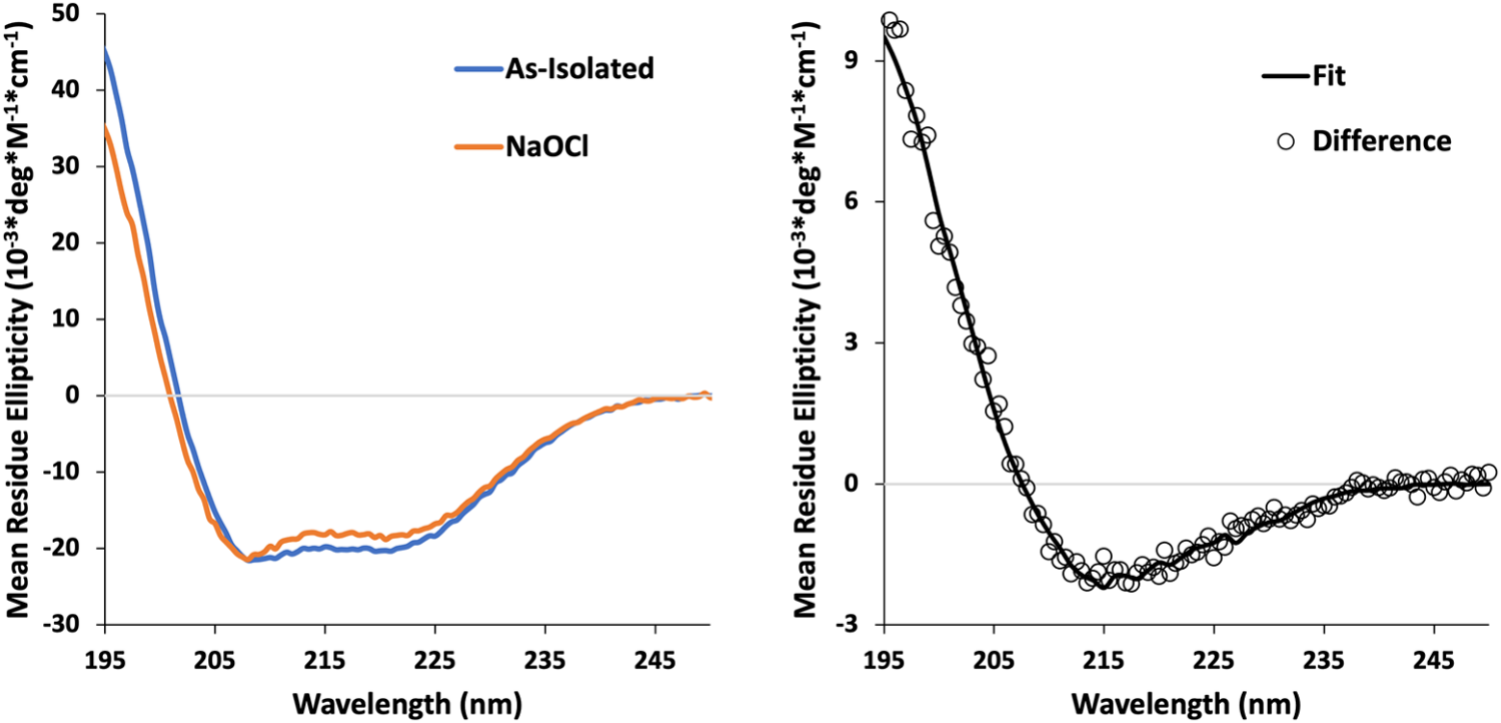
Circular dichroism (CD) spectra of *Cc* H-NOX. (A) CD spectra of the as-isolated (blue) and NaOCl-oxidized (orange) states. (B) As-isolated minus NaOCl-oxidized difference spectrum (open circles) fitted to secondary structure composition of 1.7% α helix, 45.4% antiparallel β sheet, 11.6% turn, and 41.2% other using BestSEL^31–33^ (solid line).

## Discussion

*Cc* H-NOX shares 23 to 31% sequence identity with the well-studied H-NOX proteins listed in Table 3. Like all of these H-NOX homologues except for the O_2_-sensor *Tt* H-NOX (from , since renamed ), it is selective for NO and is unable to form a stable Fe­(II)-O_2_ complex. A comparison of the vibrational spectroscopy characteristics of H-NOX family members reveals some interesting similarities and differences (Table 3). First, in every case, the resting ferrous form is 5cHS Fe­(II) with a proximal His ligand. The apparent strength of the Fe-His bond varies, with sGC exhibiting the lowest frequency for ν­(Fe-His) at 204 cm^–1^ and *Vc* H-NOX exhibiting the highest frequency at 224 cm^–1^. However, a weak Fe-His bond does not correlate with NO selectivity over O_2_ nor a preference for 5cLS over 6cLS Fe­(II)-NO complexes, as is evident from comparison between *Vc* H-NOX and *Tt* H-NOX

**Table 3:** Heme-Ligand Vibrational Mode Frequencies (cm^–1^) for H-NOX Homologues^a^,^b^,^c^

protein	ν(Fe-His)	ν(Fe-CO)	ν(C–O)	ν(Fe-NO)	ν(N–O)	refs
*Cc* H-NOX	212	481/495	1988	532	ND	this work
Vc H-NOX	224	491	1985	523	1674	^48,49^
So H-NOX	213	494	1984	ND	ND	^50^
Ns H-NOX	209	ND	ND	528, 559^d^	ND	^50,51^
Lp H-NOX 1	219	ND	ND	522	ND	^50,51^
Lp H-NOX 2	219	ND	ND	521, 550^d^	ND	^51^
Tt H-NOX^e^	217	490	1989	553	1655^d^	^48,49^
sGC	204	**473**/487	1969/**1985**	525	1677	^52–54^
sGC β1 (1–385)	206	**478**/494	1964/**1987**	525	1676	^55–57^
sGC β1 (1–194)	208	477/496	1968	526	1677	^58^

a Species names are abbreviated as follows: *Cc*, Caulobacter crescentus; *Vc*, Vibrio cholerae; *So*, Shewanella oneidensis; *Ns*, Nostoc sp; *Lp*, Legionella pneumophila; *Tt*, Thermoanaerobacter tencongensis.

b Bolded values represent the dominant conformer in mixtures. ND = Not Determined.

c ND = Not Determined.

d Indicates a 6cLS Fe­(II)-NO complex.

e Forms a stable Fe­(II)-O2 and is predicted to be an O2-sensor.

A crystal structure of *Tt* H-NOX revealed the presence of a Tyr in the distal pocket that stabilizes a hydrogen bond network around bound O_2_,^2^ presumably stabilizing the Fe­(II)-O_2_ complex. Indeed, the distal Tyr is conserved only in predicted O_2_-sensing H-NOX homologues, where it is replaced by a hydrophobic residue in NO-sensing H-NOXs. Further, mutation of the Tyr to Phe in *Tt* H-NOX decreases its O_2_ affinity, whereas introduction of a Tyr into the distal pockets of *Lp* H-NOX 2 or sGC β1 (1–385) allows formation of a weak Fe­(II)-O_2_ complex.^42^ Thus, the presence of a distal hydrogen bond donor in the form of Tyr seems to be the primary determinant of O_2_ selectivity in the H-NOX family, although other factors such as heme distortion, protein dynamics, and heme accessibility are likely to further influence ligand affinity.^43–47^

A spectroscopic hallmark of the H-NOX family is the unusually high frequency of ν­(C–O). Backbonding from filled Fe d-orbitals into empty CO π* orbitals in Fe­(II)-CO heme complexes leads to a negative correlation between ν­(C–O) and ν­(Fe-CO) and provides a useful probe of distal pocket polarity (Figure 8).^59^ H-NOX proteins inhabit the extreme end of this correlation curve, suggesting a purely hydrophobic or negatively polar distal environment that minimizes backbonding. Interestingly, *Cc* H-NOX exhibits two distinct ν­(Fe-CO) modes, indicating two CO conformers, as has been observed in sGC, its truncation mutants (Table 3), and various *Tt* H-NOX mutants.^50^ However, only a single ν­(C–O) is observed at 1988 cm^–1^ in *Cc* H-NOX. While it is possible that we are missing a second ν­(C–O) peak because that conformer is not photolabile, plotting the two conformers assuming they share a ν­(C–O) frequency shows that they are both in the range of values obtained for H-NOX relatives (Figure 8).

**Figure 8:**
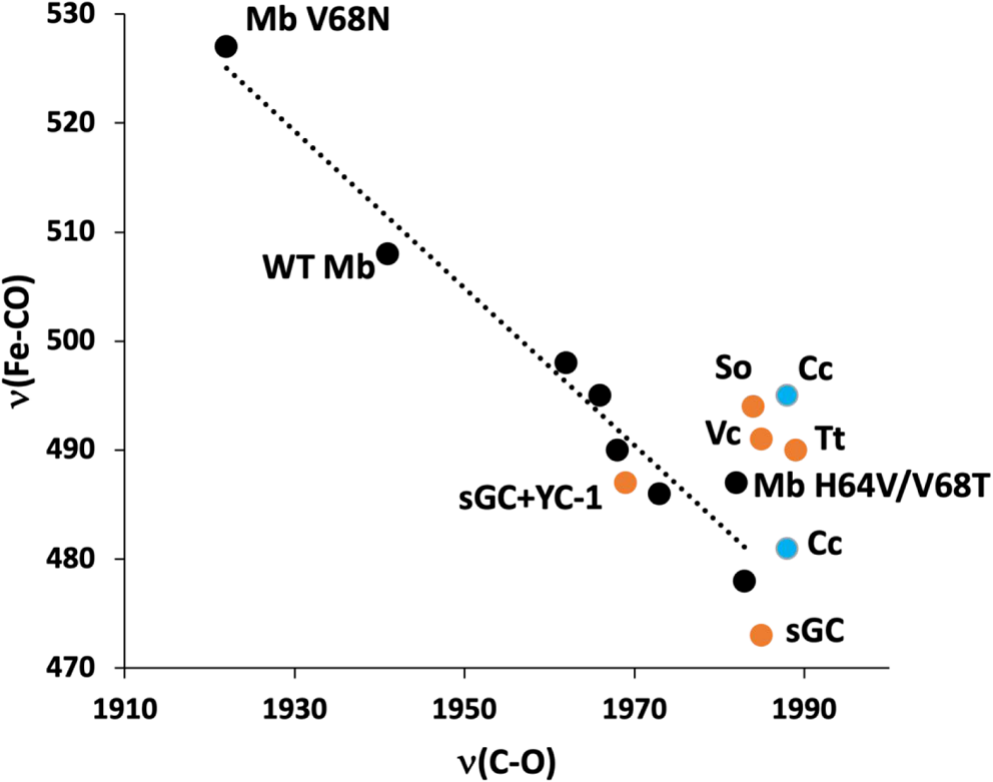
Heme-CO frequencies of histidine-ligated heme proteins. *Cc* H-NOX frequencies are indicated in blue, assuming that the 1988 cm^–1^ ν­(C–O) frequency is common to both ν­(Fe-CO) frequencies. All other H-NOX protein frequencies are indicated in orange and labeled as in Table 3. Other frequencies are indicated in black and belong to WT^65^ and mutant myoglobins (Mb)^66^ and the heme-based gas sensors FixL,^67,68^ *Ec*DOS,^69^ *Bs* HemAT,^70,71^ and CooA^72,73^ as assembled and described in Liu and Kinkaid.^74^ The correlation line was plotted without H-NOX frequencies.

In sGC, the relative proportion of CO conformers is switched in the presence of GC activators YC-1 and BAY 41–2272 to favor the state with increased backbonding at ν­(Fe-CO) ∼487 and ν­(C–O) ∼1969.^53,60,61^ Thus, the binding of these molecules perturbs the structure of the heme environment. Alterations in the CO conformers are also observed for sGC truncation constructs (Table 3), suggesting that intra- and interdomain interactions also modulate the heme environment. Strong hydrogen bond interactions between heme propionates and the absolutely conserved Y × S × R motif of H-NOX domains have been proposed to lead to the unusually weak backbonding observed for the H-NOX family.^53,62^ YC-1 binding or intra- and interdomain interactions may disrupt these bonds, leading to increased Fe-CO backbonding and activation of sGC. Recent cryo-electron microscopy structures of full-length sGC have shown that YC-1 binds very near the β-subunit heme. However, sGC structures with and without YC-1 have only been determined in the active 5cLS Fe­(II)-NO form, and the resolution precludes confident determination of subtle changes in propionate hydrogen bond interactions.^63,64^ In any case, it is noteworthy that *Cc* H-NOX is the first WT bacterial H-NOX protein, to our knowledge, to exhibit two CO conformers with ν­(Fe-CO) frequencies very similar to those observed for sGC. Although the specific interactions engaged by each conformer are unknown, their frequencies are likely a useful probe, and it will be interesting to determine whether they are sensitive to interactions with putative binding partners such as HnoK.

The UV–vis and EPR spectra of *Cc* H-NOX in the Fe­(II)-NO state, as well as the ν­(Fe-NO) frequency, are indicative of a 5cLS Fe­(II)-NO complex. Activation of H-NOX and sGC is proposed to go through a transient 6cLS Fe­(II)-NO, leading to rupture of the Fe-His bond and formation of a 5cLS Fe­(II)-NO active state.^45,75,76^ However, several H-NOX homologues exhibit some stable formation of 6cLS Fe­(II)-NO states (Table 3). In particular, *Ns* H-NOX exhibits a considerable proportion of 6cLS Fe­(II)-NO even at high NO concentrations.^77^ It also undergoes autoxidation of the Fe­(II) state in air to form a 6cLS Fe­(III) state, with EPR spectroscopy indicating either a His or Met residue as the sixth ligand. This led the authors to theorize that *Ns* H-NOX may function as a redox sensor rather than a direct NO sensor. However, the relative activities of *Ns* H-NOX in various heme states could not be evaluated, since the interaction partner of this protein is not known.

In contrast, we have shown that *Cc* H-NOX inhibits HnoK autophosphorylation. Remarkably, the 5cLS Fe­(II)-NO state of this protein is completely noninhibitory. To the best of our knowledge, this is unprecedented in the NO-selective H-NOX family. Rather, activation of *Cc* H-NOX depends on oxidation. Treatment with NaOCl results in both the complete oxidation of Cys thiols and a conversion of the heme from a 5cHS to 6cLS state, whose UV–vis spectroscopic features closely resemble those of the autoxidized *Ns* H-NOX. Treatment with diamide has no effect on the 5cHS Fe­(III) heme, but it is able to partially oxidize Cys thiols and partially activate the H-NOX for HnoK inhibition. Although other Cys modifications are possible, we hypothesize that NaOCl-oxidized H-NOX has two disulfide bonds between its 4 Cys residues (Figure 9), as has been observed for a heme-free preparation of *Vc* H-NOX,^10^ while diamide generates only a single disulfide bond and partial inhibitory activity. However, we cannot rule out the possibility of another reversible modification, such as Cys sulfenic acid (SOH), which has been shown to activate chaperone activity in Hsp33 ^78^ and to have a regulatory role in many other proteins and enzymes.^79^ Careful analysis by mass spectrometry is required to differentiate these possibilities.

**Figure 9:**
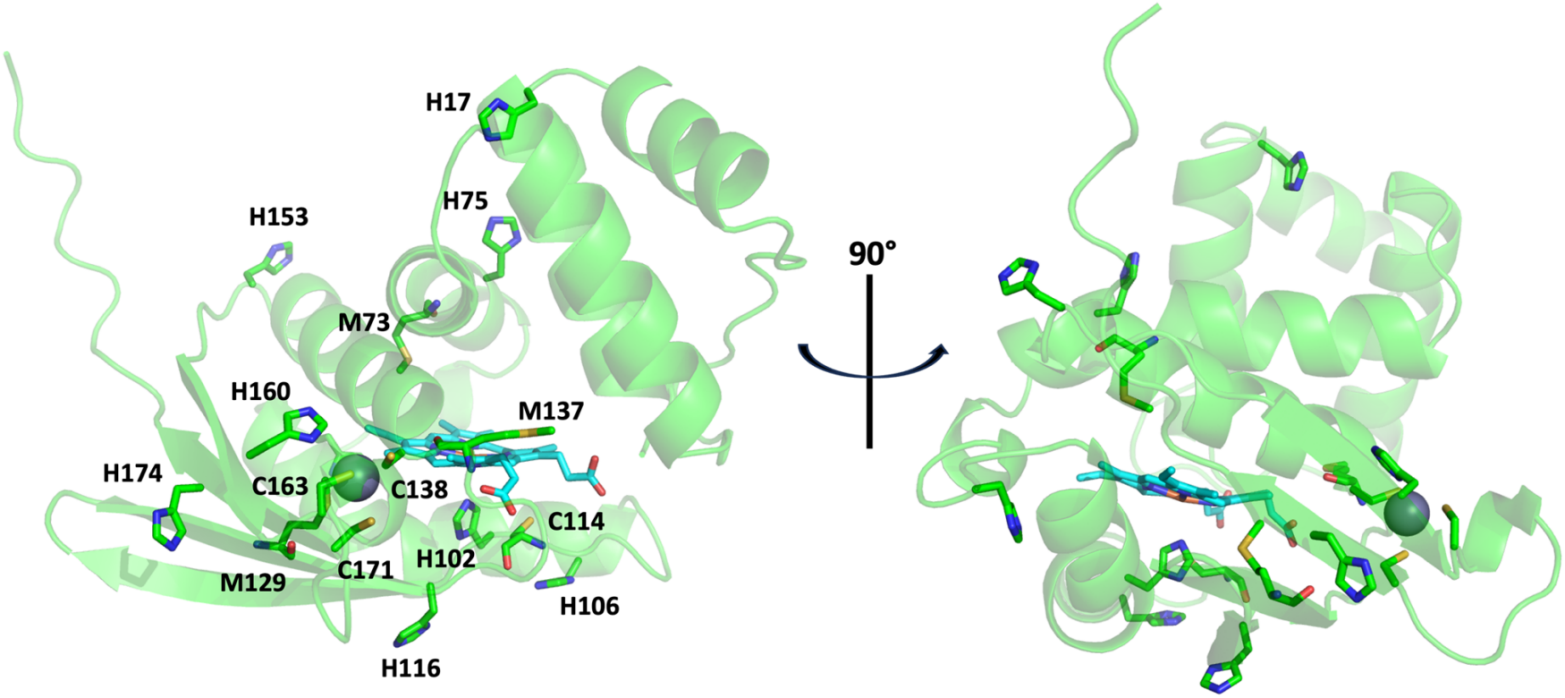
AlphaFold structure of *Cc* H-NOX. Cys, His, and Met residues are highlighted as sticks. The heme (blue sticks) and zinc ion (gray sphere) are taken from alignment with the *So* Fe­(II) H-NOX structure (PDB ID 4U99).^11^

Also, like *Vc* H-NOX, zinc is not lost from the protein upon oxidation. Rather, the coordination environment changes from three Cys S and 1 O/N, likely from the conserved His 160, to a purely N/O environment.^12^ The primary difference in the case of *Cc* H-NOX is that EXAFS data reveal strong multiple scattering contributions consistent with 4-His ligation in the oxidized state, whereas these were substantially dampened in oxidized *Vc* H-NOX, which was fit to 4 N/O ligands and 1 His. Unfortunately, an N-terminal 6-His tag was required to isolate sufficient quantities of *Cc* H-NOX for EXAFS, and proteolytic cleavage of the tag had proven ineffective. Thus, we cannot rule out complete or partial coordination of the displaced zinc in the oxidized state by the 6-His tag. However, we find this unlikely because high-affinity zinc binding to the tag would preclude binding of the protein to the nickel column. Further, we see no 4-His coordination of zinc in the reduced protein, nor do we see any reduction of zinc levels after oxidation and desalting. If the 6-His tag had sufficient affinity for zinc to acquire all of what is displaced due to oxidation, it seems that it would also have acquired zinc during expression in rich media, which would preclude purification by immobilized metal affinity chromatography (IMAC).

A survey of His residue positions in the AlphaFold structure taken from the *Cc* H-NOX Uniprot page^80^ does not reveal any obvious preformed zinc sites (Figure 9). Thus, binding of the zinc ion at a 4-His site would require substantial rearrangements of the protein backbone. Similarly, large rearrangements would be required for coordination of the heme by a second endogenous ligand, most likely a His or Met residue, given the similarity of the oxidized state UV–vis spectrum of *Cc* H-NOX to that of the autoxidized form of *Ns* H-NOX. In *Ns* H-NOX, distal coordination of the heme by a His or Met residue, as suggested by EPR spectra, would require these residues to move ∼18 Å or 8 Å, respectively, from their positions in the Fe­(II)-NO and Fe­(II)-CO structures.^77,81–83^ CD spectra suggest considerable changes in the secondary structure that may be consistent with this. It is noteworthy that secondary structure losses in *Cc* H-NOX seem to be from β sheet structures, where the zinc is likely bound in the reduced state, and two additional His residues are within 15 Å (Figure 9). These results also suggest why NaOCl is more effective at H-NOX oxidation and activation than diamide, since it can act as both a denaturant and an oxidant.^84^ More detailed structural studies of reduced and oxidized *Cc* H-NOX will be required to elucidate the structural changes accompanying oxidation, and these are underway in our laboratories.

## Conclusions

In conclusion, we have spectroscopically characterized the H-NOX protein of *C. crescentus*, the first of its group to be studied *in vitro*. For the most part, spectroscopic signatures are comparable to other H-NOX homologues except for the existence of two CO conformers, which have only been observed in a WT H-NOX domain in sGC. Despite forming a 5cLS Fe­(II)-NO species upon exposure to NO, this form is completely inactive in a HnoK autophosphorylation inhibition assay. However, full or partial oxidation of Cys residues using NaOCl or diamide, respectively, resulted in inhibitory activity proportional to the degree of Cys oxidation. NaOCl treatment also converted the heme to a 6cLS Fe­(III) species with UV–vis spectroscopic characteristics consistent with those of two endogenous protein ligands. Despite complete oxidation of Cys residues by NaOCl, zinc was retained in the oxidized state. Zinc K-edge EXAFS further revealed a change in zinc ligation from a 3 S, 1N/O to a purely N/O multiple scattering environment, best fit as 4-His ligands. Both changes would require a substantial structural rearrangement upon activation. Taken together, these data provide the first example to our knowledge of a H-NOX protein of the NO-selective group that does not respond functionally to NO and expand our understanding of the diverse functions and mechanisms manifest in this widespread protein family.

## Supplementary Material





## Data Availability

The authors agree to make any materials, data, and associated protocols relating to their published research available to researchers or readers’ requests without undue delay or qualifications.

## Notes

Sequence alignment; UV–vis spectra; and autophosphorylation data (PDF)
Plasmid sequences (ZIP)

## Abbreviations

NO: nitric oxide
H-NOX: heme nitric oxide/oxygen binding
cGMP: cyclic guanosine monophosphate
MCP: methyl-accepting chemotaxis protein
HK: histidine kinase
c-di-GMP: cyclic diguanosine monophosphate
HaCE: cyclic-di-GMP processing enzymes
HqsK: H-NOX-associated quorum-sensing kinase
RR: response regulator
rR: resonance Raman
PCR: polymerase chain reaction
MBP: maltose-binding protein
IPTG: isopropyl β-d-1-thiogalactopyranoside
Ni-NTA: nickel nitriloacetic acid
AAA: amino acid analysis
DEA-NONOate: diethylamine NONOate
DTT: dithiothreitol
CCD: charge-coupled device
CO: carbon monoxide
EPR: electron paramagnetic resonance
ICP-OES: inductively coupled plasma-optical emission spectroscopy
EDTA: ethylenediaminetetraacetic acid
DTNB: 5,5′-dithio-bis-[2-nitrobenzoic acid]
μCi: microcurie
ATP: adenosine triphosphate
HEPES: 4-(2-hydroxyethyl)-1-piperazineethanesulfonic acid
SDS–PAGE: sodium dodecyl sulfate–polyacrylamide gel electrophoresis
EXAFS: extended X-ray absorption fine structure
CD: circular dichroism
6cLS: 6-coordinate low-spin
5cLS: 5-coordinate low-spin
5cHS: 5-coordinate high-spin
FTIR: Fourier transform infrared
sGC: soluble guanylate cyclase

## References

[1] Boon E. M., Marletta M. A. (2005). Ligand specificity
of H-NOX domains: from sGC to bacterial
NO sensors. J. Inorg. Biochem.

[2] Pellicena P., Karow D. S., Boon E. M., Marletta M. A., Kuriyan J. (2004). Crystal structure
of an oxygen-binding heme domain related to soluble guanylate cyclases. Proc. Natl. Acad. Sci. U.S.A..

[3] Carlson H. K., Vance R. E., Marletta M. A. (2010). H-NOX regulation
of c-di-GMP metabolism
and biofilm formation in Legionella pneumophila. Mol. Microbiol..

[4] Liu N., Xu Y., Hossain S., Huang N., Coursolle D., Gralnick J. A., Boon E. M. (2012). Nitric
oxide regulation of cyclic
di-GMP synthesis and hydrolysis in Shewanella woodyi. Biochemistry.

[5] Plate L., Marletta M. A. (2012). Nitric oxide modulates
bacterial biofilm formation
through a multicomponent cyclic-di-GMP signaling network. Mol. Cell.

[6] Kumar S., Spiro S. (2017). Environmental and Genetic Determinants
of Biofilm Formation in Paracoccus
denitrificans. mSphere.

[7] Wang Y., Dufour Y. S., Carlson H. K., Donohue T. J., Marletta M. A., Ruby E. G. (2010). H-NOX-mediated nitric
oxide sensing modulates symbiotic
colonization by Vibrio fischeri. Proc. Natl.
Acad. Sci. U.S.A..

[8] Henares B. M., Higgins K. E., Boon E. M. (2012). Discovery
of a nitric oxide responsive
quorum sensing circuit in. ACS Chem. Biol..

[9] Price M. S., Chao L. Y., Marletta M. A. (2007). MR-1 H-NOX regulation of a
histidine kinase by nitric oxide. Biochemistry.

[10] Mukhopadyay R., Sudasinghe N., Schaub T., Yukl E. T. (2016). Heme-independent
Redox Sensing by the Heme-Nitric Oxide/Oxygen-binding Protein (H-NOX)
from. J. Biol. Chem..

[11] Herzik M. A., Jonnalagadda R., Kuriyan J., Marletta M. A. (2014). Structural
insights into the role of iron-histidine bond cleavage in nitric oxide-induced
activation of H-NOX gas sensor proteins. Proc.
Natl. Acad. Sci. U.S.A..

[12] Mukhopadhyay R., Chacón K. N., Jarvis J. M., Talipov M. R., Yukl E. T. (2020). Structural
insights into the mechanism of oxidative activation of heme-free H-NOX
from. Biochem. J..

[13] Poindexter J. S. (1981). The caulobacters:
ubiquitous unusual bacteria. Microbiol. Rev..

[14] Lee-Lopez C., Islam M. S., Meléndez A. B., Yukl E. T. (2023). Influence of the
Heme Nitric Oxide/Oxygen Binding Protein (H-NOX) on Cell Cycle Regulation
in Caulobacter crescentus. Mol. Cell. Proteomics.

[15] Ueno T., Fischer J. T., Boon E. M. (2019). Nitric Oxide Enters Quorum Sensing
via the H-NOX Signaling Pathway in Vibrio Parahaemolyticus. Front. Microbiol..

[16] Fu J., Hall S., Boon E. M. (2021). Recent evidence for multifactorial
biofilm regulation by heme sensor proteins NosP and H-NOX. Chem. Lett..

[17] Williams D. E., Nisbett L. M., Bacon B., Boon E. (2018). Bacterial Heme-Based
Sensors of Nitric Oxide. Antioxid. Redox Signaling.

[18] Lori C., Ozaki S., Steiner S., Böhm R., Abel S., Dubey B. N., Schirmer T., Hiller S., Jenal U. (2015). Cyclic di-GMP acts as a cell cycle oscillator to drive chromosome
replication. Nature.

[19] Gibson D. G., Young L., Chuang R.-Y., Venter J. C., Hutchison C. A., Smith H. O. (2009). Enzymatic assembly
of DNA molecules up to several hundred
kilobases. Nat. Methods.

[20] Bradford M. M. (1976). A rapid
and sensitive method for the quantitation of microgram quantities
of protein utilizing the principle of protein-dye binding. Anal. Biochem..

[21] Morris J. C. (1966). The Acid
Ionization Constant of HOCl from 5 to 35°. J. Phys. Chem. A.

[22] Paul K. G., Theorell H., Akeson A. (1953). The molar
light absorption of pyridine
ferroprotoporphyrin (pyridine haemochromogen). Acta Chem. Scand.

[23] Stoll S., Schweiger A. (2006). EasySpin,
a comprehensive software package for spectral
simulation and analysis in EPR. J. Magn. Reson..

[24] Riddles P. W., Blakeley R. L., Zerner B. (1979). Ellman’s reagent: 5,5′-dithiobis­(2-nitrobenzoic
acid)--a reexamination. Anal. Biochem..

[25] Riddles P. W., Blakeley R. L., Zerner B. (1983). Reassessment of Ellman’s reagent. Methods Enzymol..

[26] Schneider C. A., Rasband W. S., Eliceiri K. W. (2012). NIH Image to ImageJ:
25 years of
image analysis. Nat. Methods.

[27] George, G. N. ; Pickering, I. J. EXAFSPAK: A Suite of Computer Programs for Analysis of X-ray Absorption Spectra; Stanford Synchrotron Radiation Laboratory: Stanford, CA, USA, 1995.

[28] Binsted N., Hasnain S. S. (1996). State-of-the-Art
Analysis of Whole X-ray Absorption
Spectra. J. Synchrotron Radiat..

[29] Gurman S. J., Binsted N., Ross I. (1984). A rapid, exact
curved-wave theory
for EXAFS calculations. J. Phys. C: Solid State
Phys..

[30] Gurman S. J., Binsted N., Ross I. (1986). A rapid, exact, curved-wave
theory
for EXAFS calculations. II. The multiple-scattering contributions. J. Phys. C: Solid State Phys..

[31] Micsonai A., Moussong E., Wien F., Boros E., Vadaszi H., Murvai N., Lee Y. H., Molnar T., Refregiers M., Goto Y. (2022). BeStSel:
webserver for secondary structure and fold
prediction for protein CD spectroscopy. Nucleic
Acids Res..

[32] Micsonai A., Wien F., Bulyáki É., Kun J., Moussong É., Lee Y. H., Goto Y., Réfrégiers M., Kardos J. (2018). BeStSel: a web server for accurate protein secondary
structure prediction and fold recognition from the circular dichroism
spectra. Nucleic Acids Res..

[33] Micsonai A., Wien F., Kernya L., Lee Y. H., Goto Y., Réfrégiers M., Kardos J. (2015). Accurate secondary
structure prediction and fold recognition for circular dichroism spectroscopy. Proc. Natl. Acad. Sci. U.S.A..

[34] Spiro, T. G. Biological Applications of Raman Spectroscopy; Wiley, 1987.

[35] Kitagawa, T. Heme protein structure and the iron-histidine stretching mode. In Biological Applications of Raman Spectroscopy; Spiro, T. G. , Ed.; John Wiley and Sons, 1988; Vol. 3, pp 97–131.

[36] Ray G. B., Li X. Y., Ibers J. A., Sessler J. L., Spiro T. G. (1994). How far
can proteins bend the FeCO unit? Distal polar and steric effects in
heme proteins and models. J. Am. Chem. Soc..

[37] Derbyshire E. R., Gunn A., Ibrahim M., Spiro T. G., Britt R. D., Marletta M. A. (2008). Characterization of two different five-coordinate soluble
guanylate cyclase ferrous-nitrosyl complexes. Biochemistry.

[38] Wu G., Liu W., Berka V., Tsai A. L. (2013). The Selectivity of H-NOX for Gaseous Ligands Follows
the ″Sliding Scale Rule″ Hypothesis. Ligand Interactions
with both Ferrous and Ferric Vc H-NOX. Biochemistry.

[39] Uhl M. A., Miller J. F. (1996). Central role of
the BvgS receiver as a phosphorylated
intermediate in a complex two-component phosphorelay. J. Biol. Chem..

[40] McCleary W. R., Zusman D. R. (1990). Purification and
characterization of the Myxococcus
xanthus FrzE protein shows that it has autophosphorylation activity. J. Bacteriol..

[41] Gamble R. L., Coonfield M. L., Schaller G. E. (1998). Histidine kinase activity of the
ETR1 ethylene receptor from Arabidopsis. Proc.
Natl. Acad. Sci. U.S.A..

[42] Boon E. M., Huang S. H., Marletta M. A. (2005). A molecular
basis for NO selectivity
in soluble guanylate cyclase. Nat. Chem. Biol..

[43] Olea C., Boon E. M., Pellicena P., Kuriyan J., Marletta M. A. (2008). Probing
the function of heme distortion in the H-NOX family. ACS Chem. Biol..

[44] Weinert E. E., Plate L., Whited C. A., Olea C., Marletta M. A. (2010). Determinants of
ligand affinity and heme reactivity
in H-NOX domains. Angew. Chem., Int. Ed..

[45] Plate L., Marletta M. A. (2013). Nitric oxide-sensing H-NOX proteins govern bacterial
communal behavior. Trends Biochem. Sci..

[46] Weinert E. E., Phillips-Piro C. M., Tran R., Mathies R. A., Marletta M. A. (2011). Controlling
conformational flexibility of an O(2)-binding H-NOX domain. Biochemistry.

[47] Winter M. B., Herzik M. A., Kuriyan J., Marletta M. A. (2011). Tunnels modulate
ligand flux in a heme nitric oxide/oxygen binding (H-NOX) domain. Proc. Natl. Acad. Sci. U.S.A..

[48] Karow D. S., Pan D., Tran R., Pellicena P., Presley A., Mathies R. A., Marletta M. A. (2004). Spectroscopic
characterization of the soluble guanylate
cyclase-like heme domains from and Thermoanaerobacter tengcongensis. Biochemistry.

[49] Derbyshire E. R., Winter M. B., Ibrahim M., Deng S., Spiro T. G., Marletta M. A. (2011). Probing
domain interactions in soluble guanylate cyclase. Biochemistry.

[50] Tran R., Weinert E. E., Boon E. M., Mathies R. A., Marletta M. A. (2011). Determinants
of the heme-CO vibrational modes in the H-NOX family. Biochemistry.

[51] Boon E. M., Davis J. H., Tran R., Karow D. S., Huang S. H., Pan D., Miazgowicz M. M., Mathies R. A., Marletta M. A. (2006). Nitric
oxide binding to prokaryotic homologs of the soluble guanylate cyclase
beta1 H-NOX domain. J. Biol. Chem..

[52] Deinum G., Stone J. R., Babcock G. T., Marletta M. A. (1996). Binding of nitric
oxide and carbon monoxide to soluble guanylate cyclase as observed
with Resonance raman spectroscopy. Biochemistry.

[53] Ibrahim M., Derbyshire E. R., Marletta M. A., Spiro T. G. (2010). Probing soluble
guanylate cyclase activation by CO and YC-1 using resonance Raman
spectroscopy. Biochemistry.

[54] Kim S., Deinum G., Gardner M. T., Marletta M. A., Babcock G. T. (1996). Distal
Pocket Polarity in the Unusual Ligand Binding Site of Soluble Guanylate
Cyclase: Implications for the Control of •NO Binding. J. Am. Chem. Soc..

[55] Denninger J. W., Schelvis J. P., Brandish P. E., Zhao Y., Babcock G. T., Marletta M. A. (2000). Interaction of soluble
guanylate cyclase with YC-1:
kinetic and resonance Raman studies. Biochemistry.

[56] Zhao Y., Schelvis J. P., Babcock G. T., Marletta M. A. (1998). Identification of
histidine 105 in the beta1 subunit of soluble guanylate cyclase as
the heme proximal ligand. Biochemistry.

[57] Schelvis J.
P. M., Zhao Y., Marletta M. A., Babcock G. T. (1998). Resonance raman
characterization of the heme domain of soluble guanylate cyclase. Biochemistry.

[58] Karow D. S., Pan D., Davis J. H., Behrends S., Mathies R. A., Marletta M. A. (2005). Characterization
of functional heme domains from soluble guanylate cyclase. Biochemistry.

[59] Spiro T. G., Soldatova A. V., Balakrishnan G. (2013). CO, NO and O(2) as Vibrational Probes
of Heme Protein Interactions. Coord. Chem. Rev..

[60] Li Z., Pal B., Takenaka S., Tsuyama S., Kitagawa T. (2005). Resonance Raman Evidence
for the Presence of Two Heme Pocket Conformations with Varied Activities
in CO-Bound Bovine Soluble Guanylate Cyclase and Their Conversion. Biochemistry.

[61] Martin E., Czarnecki K., Jayaraman V., Murad F., Kincaid J. (2005). Resonance
Raman and Infrared Spectroscopic Studies of High-Output Forms of Human
Soluble Guanylyl Cyclase. J. Am. Chem. Soc..

[62] Xu C., Ibrahim M., Spiro T. G. (2008). DFT analysis
of axial and equatorial
effects on heme-CO vibrational modes: applications to CooA and H-NOX
heme sensor proteins. Biochemistry.

[63] Kang Y., Liu R., Wu J. X., Chen L. (2019). Structural insights into the mechanism
of human soluble guanylate cyclase. Nature.

[64] Liu R., Kang Y., Chen L. (2021). Activation mechanism of human soluble
guanylate cyclase by stimulators and activators. Nat. Commun..

[65] Li T., Quillin M. L., Phillips G. N., Olson J. S. (1994). Structural
determinants of the stretching frequency of CO bound to myoglobin. Biochemistry.

[66] Anderton C. L., Hester R. E., Moore J. N. (1997). A chemometric
analysis of the resonance
Raman spectra of mutant carbonmonoxy-myoglobins reveals the effects
of polarity. Biochim. Biophys. Acta.

[67] Rodgers K. R., Lukat-Rodgers G. S., Barron J. A. (1996). Structural basis for ligand discrimination
and response initiation in the heme-based oxygen sensor FixL. Biochemistry.

[68] Miyatake H., Mukai M., Adachi S., Nakamura H., Tamura K., Iizuka T., Shiro Y., Strange R. W., Hasnain S. S. (1999). Iron coordination
structures of oxygen sensor FixL characterized by Fe K-edge extended
x-ray absorption fine structure and resonance raman spectroscopy. J. Biol. Chem..

[69] Sato A., Sasakura Y., Sugiyama S., Sagami I., Shimizu T., Mizutani Y., Kitagawa T. (2002). Stationary
and time-resolved resonance
Raman spectra of His77 and Met95 mutants of the isolated heme domain
of a direct oxygen sensor from. J. Biol. Chem..

[70] Aono S., Kato T., Matsuki M., Nakajima H., Ohta T., Uchida T., Kitagawa T. (2002). Resonance
Raman and ligand binding
studies of the oxygen-sensing signal transducer protein HemAT from
Bacillus subtilis. J. Biol. Chem..

[71] Yoshimura H., Yoshioka S., Kobayashi K., Ohta T., Uchida T., Kubo M., Kitagawa T., Aono S. (2006). Specific hydrogen-bonding
networks responsible for selective O2 sensing of the oxygen sensor
protein HemAT from Bacillus subtilis. Biochemistry.

[72] Coyle C. M., Puranik M., Youn H., Nielsen S. B., Williams R. D., Kerby R. L., Roberts G. P., Spiro T. G. (2003). Activation mechanism
of the CO sensor CooA. Mutational and resonance Raman spectroscopic
studies. J. Biol. Chem..

[73] Vogel K. M., Spiro T. G., Shelver D., Thorsteinsson M. V., Roberts G. P. (1999). Resonance Raman evidence for a novel
charge relay activation
mechanism of the CO-dependent heme protein transcription factor CooA. Biochemistry.

[74] Liu Y., Kincaid J. R. (2021). Resonance Raman
studies of gas sensing heme proteins. J. Raman
Spectrosc..

[75] Olea C., Herzik M. A., Kuriyan J., Marletta M. A. (2010). Structural insights into the molecular mechanism of
H-NOX activation. Protein Sci..

[76] Erbil W. K., Price M. S., Wemmer D. E., Marletta M. A. (2009). A structural basis
for H-NOX signaling in by trapping a histidine kinase inhibitory conformation. Proc. Natl. Acad. Sci. U.S.A..

[77] Tsai A. L., Berka V., Martin F., Ma X., van den
Akker F., Fabian M., Olson J. S. (2010). Is Nostoc H-NOX
a NO sensor or redox switch?. Biochemistry.

[78] Ilbert M., Horst J., Ahrens S., Winter J., Graf P. C., Lilie H., Jakob U. (2007). The redox-switch
domain of Hsp33
functions as dual stress sensor. Nat. Struct.
Mol. Biol..

[79] Reddie K. G., Carroll K. S. (2008). Expanding the functional diversity of proteins through
cysteine oxidation. Curr. Opin. Chem. Biol..

[80] Varadi M., Anyango S., Deshpande M., Nair S., Natassia C., Yordanova G., Yuan D., Stroe O., Wood G., Laydon A. (2022). AlphaFold Protein Structure Database: massively expanding
the structural coverage of protein-sequence space with high-accuracy
models. Nucleic Acids Res..

[81] Ma X., Sayed N., Beuve A., van den Akker F. (2007). NO and CO
differentially activate soluble guanylyl cyclase via a heme pivot-bend
mechanism. EMBO J..

[82] Martin F., Baskaran P., Ma X., Dunten P. W., Schaefer M., Stasch J. P., Beuve A., van den Akker F. (2010). Structure
of cinaciguat (BAY 58–2667) bound to Nostoc H-NOX domain reveals
insights into heme-mimetic activation of the soluble guanylyl cyclase. J. Biol. Chem..

[83] Kumar V., Martin F., Hahn M. G., Schaefer M., Stamler J. S., Stasch J. P., van den
Akker F. (2013). Insights into BAY 60–2770
activation and S-nitrosylation-dependent desensitization of soluble
guanylyl cyclase via crystal structures of homologous nostoc H-NOX
domain complexes. Biochemistry.

[84] Winter J., Ilbert M., Graf P. C., Ozcelik D., Jakob U. (2008). Bleach activates
a redox-regulated chaperone by oxidative protein unfolding. Cell.

