# Supplementary material for: Oxidative Activation of the Heme Nitric Oxide/Oxygen-Binding Protein (H-NOX) from
Source: Biochemistry. 2025 Jul 28;64(15):3345–57. doi: 10.1021/acs.biochem.5c00262 (PMC12329717; doi:10.1021/acs.biochem.5c00262)
Supplement: Supplementary file 1 [file bi5c00262_si_001.pdf]

Supporting Information

Oxidative activation of the heme nitric oxide/oxygen binding protein (H-NOX) from *Caulobacter crescentus*

Aishat Alatishe[a§], Therese Albert[b§], Cameron Christopher Lee-Lopez[a], Rashedul Hasan[a], Pierre Moënne-Loccoz[b], Kelly N. Chacón[c], and Erik T. Yukl[a,*]

[a]Department of Chemistry and Biochemistry, New Mexico State University, Las Cruces, NM 88003.
[b]Department of Chemical Physiology and Biochemistry, School of Medicine, Oregon Health & Science University, Portland, OR 97239
[c]Department of Chemistry, Reed College, Portland, OR 97202.

---

[*]Correspondence: Tel. 575 646 3176; Fax. 575 646 2649; E-mail: etyukl@nmsu.edu

[§]These authors contributed equally to this work.

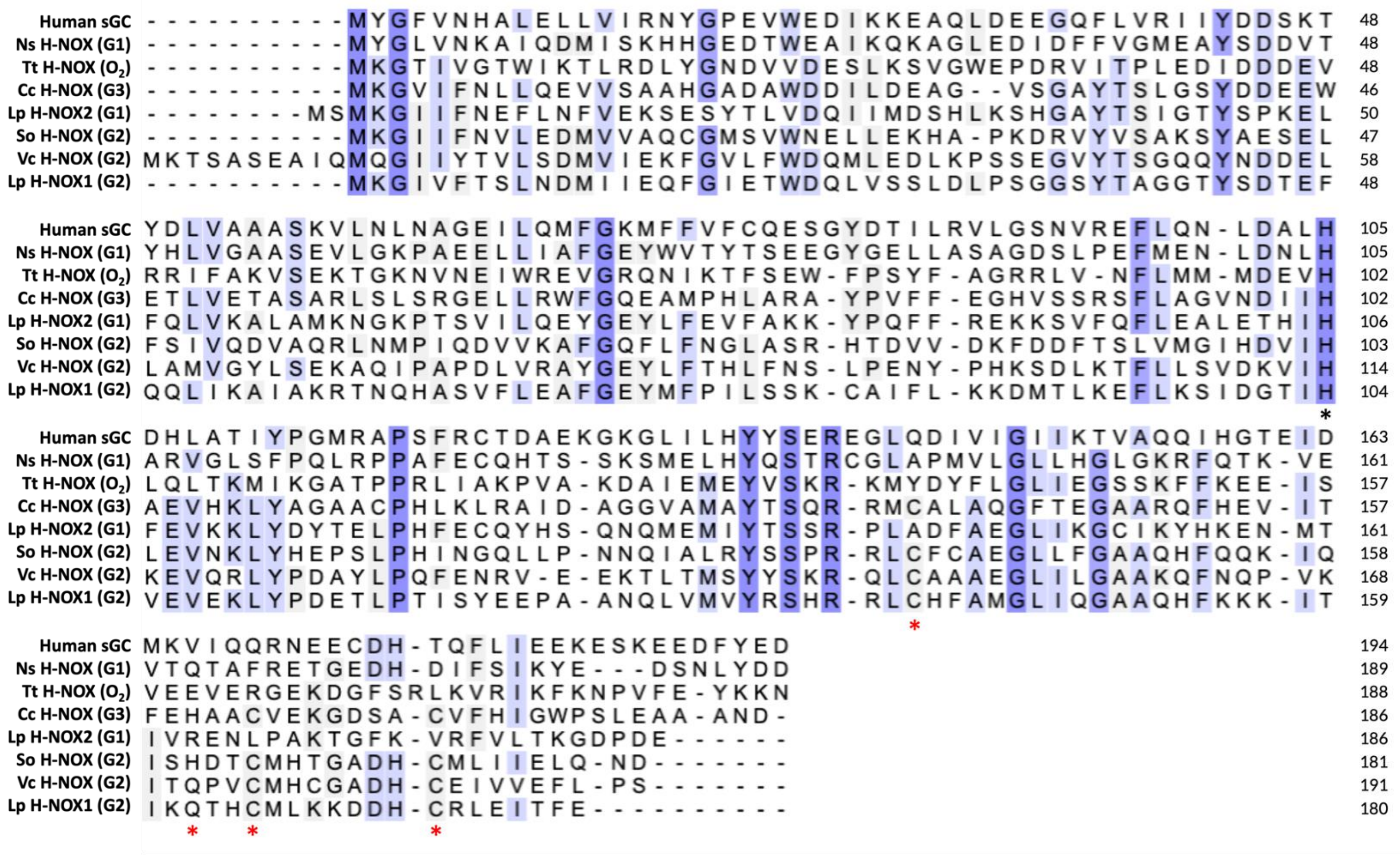

Figure S1: Multiple sequence alignment of H-NOX domains. The NO-sensitive H-NOX domain group number or if it is an O_2 sensor is given in parentheses after the protein name. Black asterisk (*) indicates the proximal ligand to the heme iron. Red asterisks (*) indicate zinc ligands in G2 and G3 H-NOX proteins. Abbreviations used are: sGC, soluble guanylate cyclase; Ns, *Nostoc sp.*; Tt, *Thermoanaerobacter tencongensis* (aka *Caldanaerobacter subterraneus* subsp. *tencongensis*); Cc, *Caulobacter crescentus* (aka *Caulobacter vibriodes*); Lp, *Legionella pneumophila*; So, *Shewanella oneidensis*; Vc, *Vibrio cholerae*.

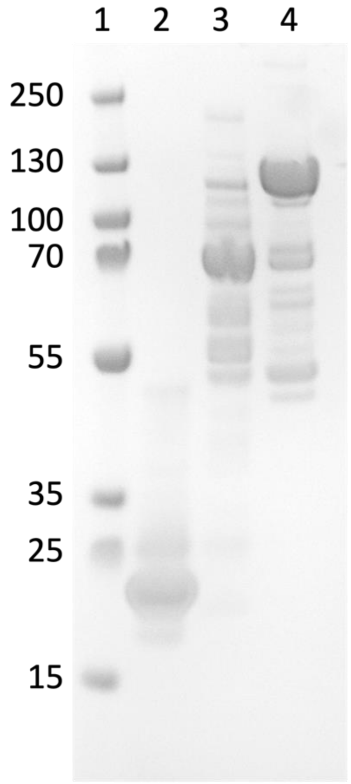

Figure S2: SDS-PAGE of proteins used in this study. Lane 1: MW ladder with masses listed in kDa. Lane 2: WT H-NOX-6His, predicted MW = 21,397. Lane 3: D543N HnoK-6His, predicted MW = 69,393. D543N HnoK-MBP, predicted MW = 109,116

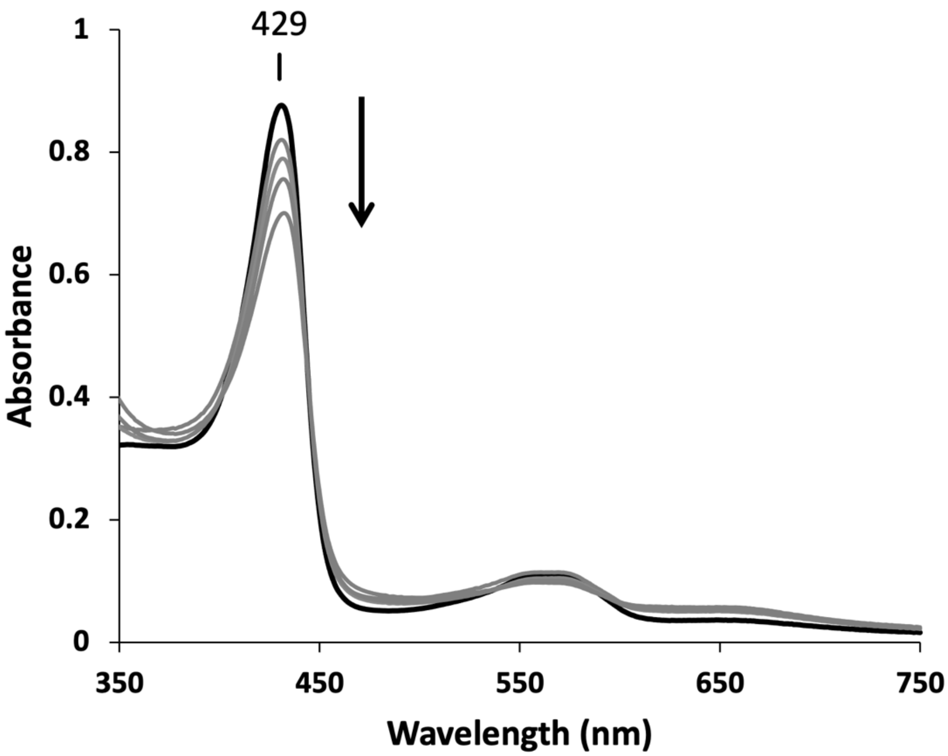

Figure S3: Oxygen sensitivity of Fe(II) *Cc* H-NOX. The Fe(II) state was formed by anaerobic incubation with 50 mM DTT in a sealed cuvette followed by exposure to air. The bold black trace is the Fe(II) state immediately before exposure while gray traces were taken at 30-minute intervals after exposure.

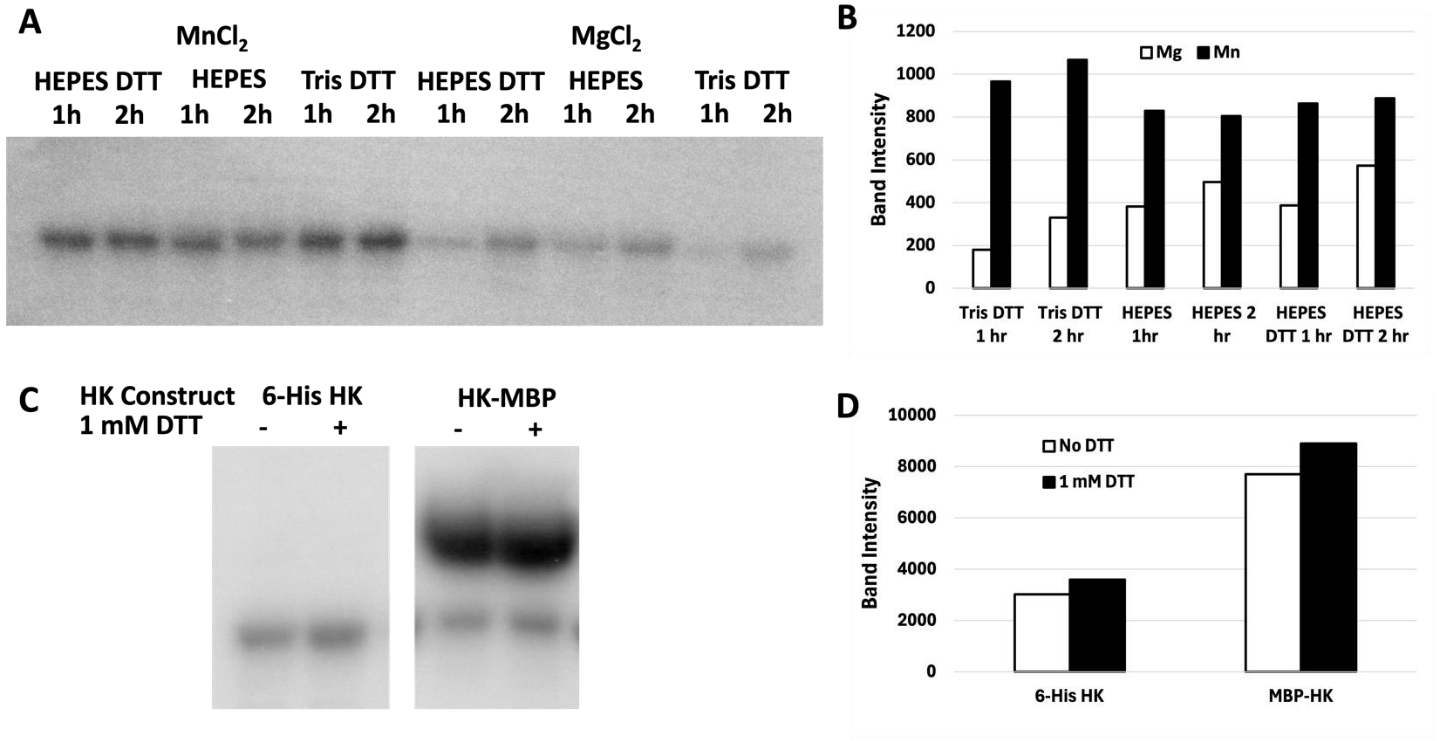

Figure S4: Autophosphorylation activity of various preparations of HnoK under different conditions in the absence of H-NOX. (A and C) Autoradiography of dried SDS-PAGE gels of 6-His HnoK (A) and a comparison of 6-His HnoK and HnoK-MBP (C). (B and D) Quantified band intensities.

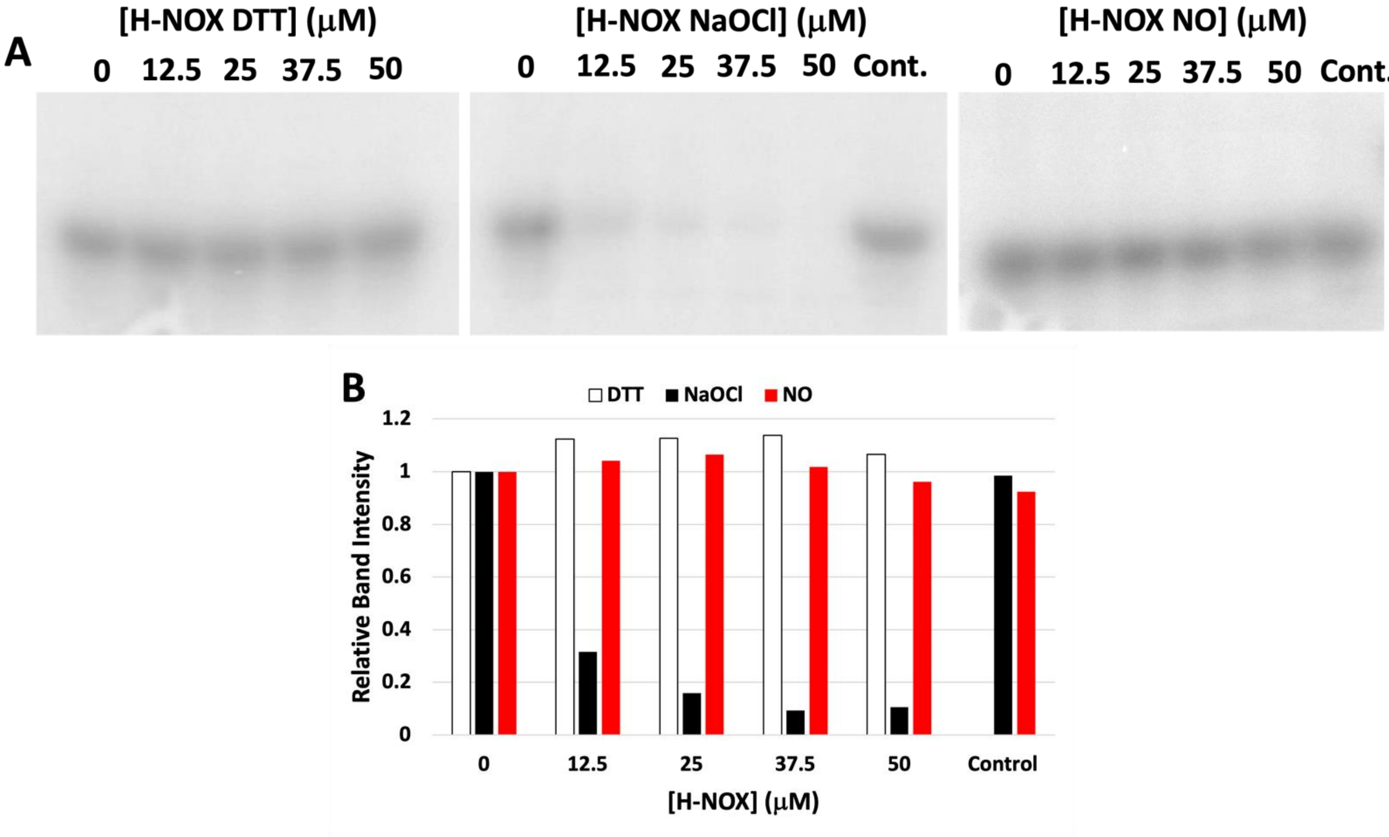

Figure S5. Inhibition of HnoK autophosphorylation by *Cc* H-NOX. (A) Representative autoradiography of 6-His HnoK autophosphorylation in the presence of increasing concentrations of H-NOX in various states. (B) Quantitation of HnoK autophosphorylation. "Control" refers to addition of DTT to 1 mM to the highest concentration of NaOCl H-NOX or addition of NONOate-treated buffer to HnoK alone in the H-NOX NO data series (see Materials and Methods).

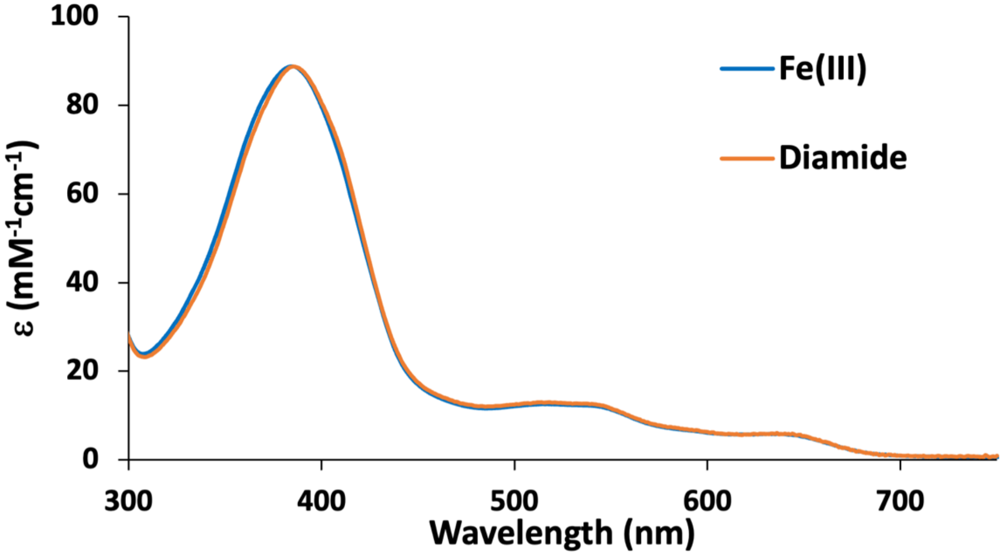

Figure S6. UV-vis spectra of Cc H-NOX as-isolated before (blue, Fe(III)) and after (orange, Diamide) addition of 1 mM diamide.

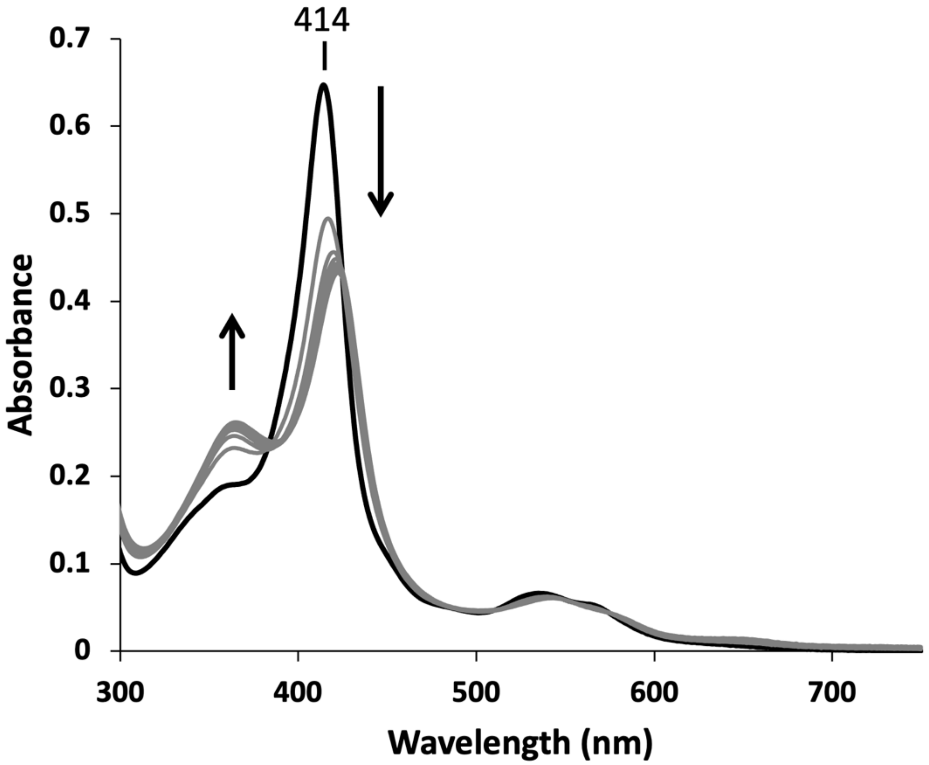

Figure S7: Reaction of *Cc* H-NOX NaOCl with 1 mM DTT. The NaOCl-oxidized state was formed by incubation with 1 mM NaOCl for 1 hr at room temperature followed by desalting and is represented in the bold black trace. Gray traces were taken at 2-minute intervals following addition of DTT to 1 mM.
